# Ligase-dependent and independent functions of the C-terminus of Mms21 contribute to optimal growth and genome stability in *Saccharomyces cerevisiae*

**DOI:** 10.1091/mbc.E25-11-0567

**Published:** 2026-04-08

**Authors:** Cheung Li, Anny Vo, Nkechinye Baadi, Yee Mon Thu

**Affiliations:** ^a^Department of Biology, Colby College, Waterville, ME 04901; University of Alberta

## Abstract

An evolutionarily conserved E3 SUMO ligase, Mms21, orchestrates genome integrity processes. Our study examined a mutant of *Saccharomyces cerevisiae* Mms21, analogous to a mutant identified in a rare human condition characterized by genome instability. The human mutation C-terminally truncated the Mms21 protein, without affecting the residues in the E3 ligase domain. Thus, we hypothesized that the C-terminus regulated ligase-independent functions of Mms21. Truncating the last 22 amino acids of yeast Mms21—designated as *mms21Δ22* mutants—mimicked the human disease mutation. *mms21Δ22* mutants exhibited slower growth and increased DNA damage sensitivity than the wild-type and two well-characterized mutants of Mms21—one with two missense mutations in the enzymatic domain and another without the entire enzymatic domain and the C-terminus. Furthermore, *mms21Δ22* mutants exhibited a G_2_–M delay during unchallenged growth. The *mms21Δ22* allele reduced Mms21 protein levels, but the phenotypes of *mms21Δ22* mutants simply could not be attributed to diminished protein levels. Our genetic data suggested that the C-terminus contributed to both ligase-dependent and -independent functions of Mms21 and opposed the activity of the adjacent domain, thereby fine-tuning genome integrity. The *mms21Δ22* disease allele analogue further enhanced our understanding of Mms21’s functions beyond its ligase activity in genome instability conditions.

## Abstract (executive summary)

- The SUMO E3 ligase function of an evolutionarily conserved Mms21 is crucial for genome integrity. The ligase-independent function of Mms21 in genome stability needs further exploration. To this end, we characterized a C-terminal truncation mutant of budding yeast Mms21 (*mms21Δ22*), analogous to a human mutation associated with genome instability conditions.
- Using genetic approaches, we demonstrated that the C-terminus regulated both ligase-dependent and -independent functions of Mms21 and restrained the activity of the adjacent domain, ensuring optimal genome stability.
- The *mms21Δ22* allele is valuable to gain new mechanistic insights into how Mms21 coordinates genome integrity pathways and associated human genome instability conditions.

## INTRODUCTION

Organisms routinely encounter genome-destabilizing conditions of varying intensity. Under such circumstances, the genome may become prone to mutations, chromosomal rearrangements, or sensitive to genotoxic stress. Organisms have evolved multiple mechanisms to tolerate, repair, and resolve aberrant structures of DNA resulting from exogenous and endogenous genotoxic stresses. Regulation through SUMO (small ubiquitin-like modifier) orchestrates many aspects of genome integrity pathways in eukaryotes. Sumoylation represents a series of biochemical reactions in which an E1 SUMO activating enzyme, an E2 SUMO conjugating enzyme, and E3 SUMO ligases coordinate to covalently attach SUMO to target proteins. Sumoylation regulates molecular processes such as protein complex formation and disassembly, proteins’ association with chromatin, and subcellular relocalization of proteins (Sarangi and Zhao, 2015; Vertegaal, 2022; Thu, 2024). In unicellular and multicellular eukaryotes, a single E1 is responsible for activating SUMO to initiate the process. A single E2, Ubc9 (ubiquitin conjugating 9) in both yeast and mammals, coordinates with E3 enzymes to conjugate SUMO to target proteins (Sarangi and Zhao, 2015; Vertegaal, 2022; Thu, 2024). An evolutionarily conserved E3 ligase, Mms21 (methyl methanesulfonate sensitivity 21), plays diverse roles in genome stability (Peng and Zhao, 2023).

**FIGURE 1:**
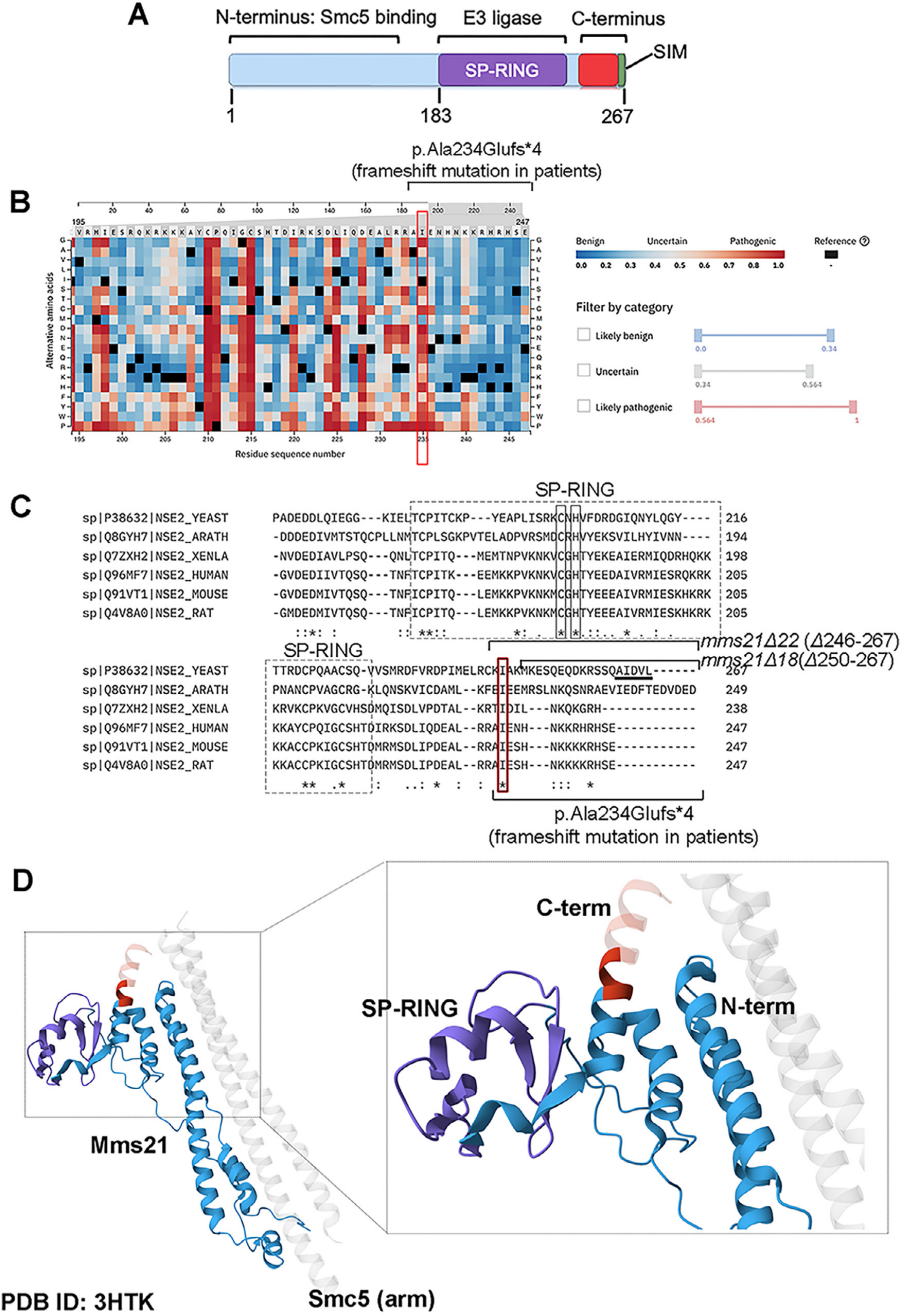
Structural analyses of Mms21 to understand *mms21Δ22* mutants, which carry the mutation analogous to the C-terminal truncation mutant of Mms21 in human patients. (**A)** Depiction of different domains of *S. cerevisiae* Mms21. The N-terminus of Mms21 interacts with Smc5, and this interaction is crucial for resisting DNA damage (Duan *et al.*, 2009). The SP-RING is responsible for the E3 SUMO ligase activity of Mms21 (Andrews *et al.*, 2005; Zhao *et al.*, 2005; Peng *et al.*, 2023). SUMO-interacting motif (SIM) at the end of *S. cerevisiae* Mms21 enhances E3 SUMO ligase activity(Varejao *et al.*, 2021). For the purpose of this study, the C-terminus is defined as the last 22 amino acids of *S. cerevisiae* Mms21. The numbers represent the amino acid coordinates. (**B)** Alpha Missense analysis of the C-terminus. This region is missing in one of the alleles of the compound heterozygous *NSCME2* mutations (p.Ala234Glufs*4) associated with a rare genetic condition in human patients. Alpha Missense analysis reveals I235 (red box) to have a high pathogenicity score, a probability of a variant having a negative impact on the protein's function (Payne *et al.*, 2014). The UniProt ID of Mms21 used in Alpha Missense analysis is Q96MF7. (**C)** Multiple sequence alignment of Mms21 proteins in *S. cerevisiae* (YEAST), *Arabidopsis thaliana* (ARATH), *Xenopus laevis* (XENLA), *Homo sapiens* (HUMAN), *Mus musculus* (MOUSE), and *Rattus norvegicus* (RAT). The protein sequences were retrieved from UniProt and the alignment was performed using ClustalOmega. Only the SP-RING domain, the rest of the C-terminal end of the protein were shown. The SIM in the *S. cerevisiae* protein is underlined. The regions missing in *mms21Δ22*, *mms21Δ18*, and in the patient mutation (p.Ala234Glufs*4) are indicated. The conserved residue (I235 in the human protein) is highlighted with a red box. (**D)** The position of the C-terminus within the structure of *S. cerevisiae* Mms21 bound to Smc5 (PDB ID: 3HTK) (Duan *et al.*, 2009). This crystal structure does not contain the last nine amino acids. Red indicates the C-terminus. Dark red represents four amino acids (KIAK) present in *mms21Δ18* mutants but absent in *mms21Δ22* mutants. The SP-RING domain is highlighted by purple. The rest of Mms21 is in blue.

Mms21 is a subunit of the chromatin-associated Smc5/6 (structural maintenance of chromosome 5/6) complex that regulates DNA replication, repair, recombination, and chromosome segregation (Peng and Zhao, 2023). The N-terminus of Mms21 interacts with Smc5, and this interaction is required for cell survival and resisting DNA damage (Figure 1A) (Duan *et al.*, 2009). The SP-RING (Siz2/PIAS really interesting new gene) domain in Mms21 is responsible for the E3 ligase activity and is located closer to the C-terminal end of the protein (Figure 1A) (Zhao and Blobel, 2005; Peng and Zhao, 2023). Structurally, the SP-RING faces away from the Smc5/6 complex, and this allows the ligase domain to interact with Ubc9 and SUMO (Duan *et al.*, 2009; Varejao *et al.*, 2021; Peng and Zhao, 2023).

*MMS21* is essential, but the E3 ligase activity is dispensable for survival in both yeast and mammalian cells (Andrews *et al.*, 2005; Zhao and Blobel, 2005; Rai *et al.*, 2011; Jacome *et al.*, 2015). In *Saccharomyces cerevisiae*, mutants without Mms21’s E3 ligase activity become highly sensitive to genotoxic agents, such as methyl methane sulfonate (MMS), hydroxyurea, and doxorubicin (e.g., Zhao and Blobel, 2005; Huang *et al.*, 2007; Varejao *et al.*, 2018). Similar phenotypes have been observed in fission yeast and mammalian cells (Andrews *et al.*, 2005; Potts and Yu, 2005; Jacome *et al.*, 2015). Even in the absence of exogenous stress, *mms21* ligase-deficient mutants exhibit gross chromosomal rearrangements (Liang *et al.*, 2018). The significance of Mms21’s activity in genome integrity is further underscored by the identification of mis-regulated Mms21’s activities in human patients (e.g., Chinen *et al.*, 2014; Payne *et al.*, 2014; Pekkola Pacheco *et al.*, 2023; Zhu *et al.*, 2023).

Genetic data suggest that Mms21 limits aberrant DNA intermediates by functioning downstream of the DNA damage tolerance pathways, which can generate these structures (Branzei *et al.*, 2008; Choi *et al.*, 2010). Consistently, one hallmark of *mms21* and *smc5* mutants is the accumulation of DNA intermediates resulting from recombination or recombination-dependent pathways (Branzei *et al.*, 2006; Branzei *et al.*, 2008). Multiple mechanisms by which Mms21 coordinates DNA metabolic processes explain this phenotype. In general, the Smc5/6 complex contributes to the processing of DNA structures, some of which arise from replication stress. When faced with challenges during S-phase, cells can utilize multiple pathways to resume replication promptly (Szakal *et al.*, 2024; Fiedorowicz *et al.*, 2025). The error-free template switching pathway serves as a mechanism to bypass the DNA lesion on the template but creates DNA intermediates that require the function of the STR complex consisting of Sgs1 (slow growth suppressor 1), Top3 (topoisomerase 3), DNA topoisomerase, and Rmi1 (RecQ mediated genome instability 1) in a process called dissolution (Bizard and Hickson, 2014). The Smc5/6/Mms21 complex enhances the dissolution activity of the STR complex by promoting Sgs1 sumoylation, a process important for STR complex dynamics (Bermudez-Lopez *et al.*, 2016; Bonner *et al.*, 2016; Bermudez-Lopez and Aragon, 2017). Another pathway to remedy replication fork stalling includes fork regression, a process in which two newly synthesized strands anneal with each other to reverse the fork (Meng and Zhao, 2017). The Smc5/6 complex can limit the activity of Mph1, a helicase that can mediate fork regression (Chen *et al.*, 2009; Xue *et al.*, 2014; Zapatka *et al.*, 2019).

The essential function of the Smc5/6 and Mms21 complex can be attributed to its role in G_2_–M. Using cells that expressed Mms21 in S-phase only, Menolfi *et al.* demonstrated that the G_2_–M function of the Smc5/6 complex and Mms21 is crucial for preventing accumulation of DNA structures arising from endogenous replication stress and replication through natural pause sites (Menolfi *et al.*, 2015). Their data are further corroborated by the observation that the Smc5/6/Mms21 complex associates with chromatin regions that are difficult to replicate (Torres-Rosell *et al.*, 2005; Takahashi *et al.*, 2008; Menolfi *et al.*, 2015; Agashe *et al.*, 2021). The literature to date collectively suggests that Mms21 contributes to diverse mechanisms to relieve replication stress and genome instability through its E3 ligase activity. Consistently, a proteomic study revealed multiple DNA replication and repair proteins to be targets of Mms21, suggesting that the E3 ligase function of Mms21 plays a prominent role in genome instability (Albuquerque *et al.*, 2013).

Indeed, much of our understanding of Mms21’s function is based on characterizing the phenotypes of *mms21-CH* and *mms21-11* alleles in *S. cerevisiae*, both of which are deficient in the ligase activity (Andrews *et al.*, 2005; Zhao and Blobel, 2005). Nevertheless, given the diverse roles of Mms21 and its functional interactions with multiple pathways, it is conceivable that different regions of the protein coordinate to augment the function of Mms21 in both ligase-dependent and -independent manners. In a human rare genetic condition, an *NSMCE2* (the human gene of *MMS21*) variant designated as p.Ala234Glufs*4 was identified (Payne *et al.*, 2014). This mutation truncates the C-terminus of the human Mms21 protein but leaves the E3 ligase domain of Mms21 intact (Figure 1A–C) (Payne *et al.*, 2014). However, the mutant cannot fully complement the loss of Mms21 in zebrafish, suggesting that the truncation alters the function of Mms21 (Payne *et al.*, 2014). This mutation was identified as one of the compound heterozygous mutations, along with another *NSMCE2* allele that encodes a mutant protein lacking the SP-RING and the entire C-terminus (Payne *et al.*, 2014). Cells with *NSMCE2* compound heterozygous mutations exhibited an increase in sister chromatid exchange and micronuclei formation, suggesting that these *NSMCE2* variants lead to high genome instability (Payne *et al.*, 2014). Patients with these compound heterozygous mutations exhibited developmental delay and insulin resistance (Payne *et al.*, 2014).

We reasoned that a closer characterization of the C-terminus could provide additional mechanistic insights into ligase-independent functions. To this end, we modeled the p.Ala234Glufs*4 human mutation from the study by Payne *et al.* in *S. cerevisiae* using the yeast gene *MMS21* (Payne *et al.*, 2014). In addition, using yeast as a haploid and an easily tractable model organism allowed us to dissect the genetic interactions of the mutant. We found that a functional C-terminus (defined as the last 22 amino acids of the *S. cerevisiae* Mms21 protein) was crucial for growth under unchallenged as well as DNA damage conditions. Intriguingly, cells without the C-terminus grew worse and were more sensitive to DNA damage than well-characterized ligase-deficient *mms21* mutants. This phenotype could be explained by our observations that the C-terminal truncation mutant impaired both ligase-dependent and -independent functions. Furthermore, we provided genetic evidence to support the idea that the C-terminus counteracted the function of the adjacent region containing the SP-RING domain to fine-tune genome stability processes.

## RESULTS

The human p.Ala234Glufs*4 *NSMCE2* mutation associated with a rare genetic condition resulted in a frameshift mutation (Payne *et al.*, 2014). The resulting mutant human protein was missing the last 14 amino acids within the C-terminus, but this frameshift mutation did not affect the sequences within the SP-RING domain of Mms21 (Figure 1, B and C) (Payne *et al.*, 2014). To predict whether the C-terminal truncation mutant of *NSMCE2* identified in patients affected Mms21’s activity, we performed Alpha Missense analysis. This analysis predicted one amino acid within the C-terminus of human Mms21 (UniProt ID Q96MF7), I235, to have a high pathogenicity score for most amino acid changes, suggesting that a mutation in this position is likely to disrupt the function of the protein (Figure 1B). The pathogenicity score is defined as the probability of a variant being detrimental to the protein. To understand the functional significance of this region, we introduced the analogous truncation into *S. cerevisiae* Mms21.

### *mms21Δ22*, but not *mms21Δ18 or mms21sl,* mutants are compromised for growth and sensitive to DNA damage

**FIGURE 2:**
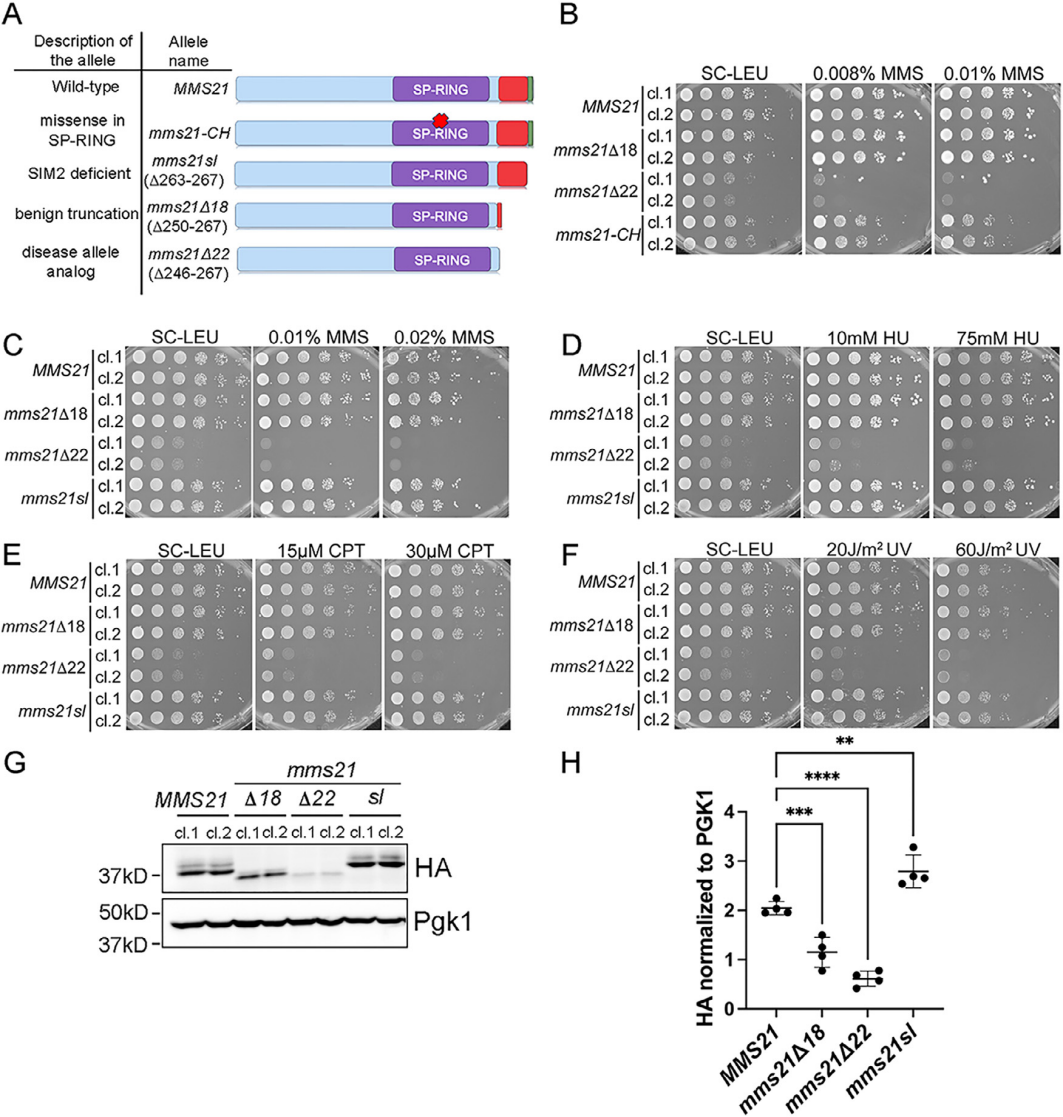
*mms21Δ22* mutants exhibit slow growth and high sensitivity to different types of genotoxic stress. **(A)** Different mutants of Mms21 are used in the Figure, and a description of each allele. **(B−F)** Successive 10-fold serial dilutions of the indicated strains were grown on SC-LEU plates without or with different types of agents that compromise genome stability. (**B)** *mms21Δ22* mutants, but not *mms21Δ18* mutants, grew slower than wild-type and *mms21-CH,* and were highly sensitive to MMS (*n* = 2 for each clone). (**C)** *mms21Δ22* mutants, but not *mms21Δ18* or *mms21sl* mutants, grew slower than wild-type and were highly sensitive to MMS (*n* = 3 for each clone). (**D−F)** *mms21Δ22* mutants, but not *mms21Δ18* or *mms21sl* mutants, were highly sensitive to hydroxyurea (HU) (**D**), camptothecin (CPT) (**E**), and ultraviolet radiation (UV) (**F**) (*n* = 2 for each clone). (**G)** Western blot analysis of the indicated strains in which Mms21 wild-type or mutant proteins were epitope tagged with 3HA at the C-terminus and detected with an HA antibody. Pgk1 was used as a loading control. (**H)** Quantification of western blots (*n* = 4). Error bars represent SDs. One-way ANOVA was performed to determine the statistical significance (**0.003, ***0.0007, ****<0.0001) (cl. = clones)

Based on multispecies sequence alignment using Clustal Omega, we generated two yeast Mms21 C-terminal truncation mutants—*mms21Δ18* and *mms21Δ22* mutants, missing the last 18 and 22 amino acids, respectively (Figure 1C). Using the published crystal structure of *S. cerevisiae* Mms21 bound to Smc5 (PDB ID: 3HTK), we visualized the location of those truncated residues within the structure (Duan *et al.*, 2009). The last 18 amino acids extend outwards from the core of the protein, ostensibly not accessible for intramolecular interactions with other regions of the protein (light red, Figure 1D). The four amino acids (“KIAK”) present in the *mms21Δ18* mutant but not in the *mms21Δ22* mutant are flanked by the N-terminus and the SP-RING domain (dark red, Figure 1D). Structural information suggested that truncation of 22 amino acids was more likely to have an impact on the function of the protein. We used a well-characterized mutant, *mms21-CH*, as a control, which harbors two missense mutations in the SP-RING domain and is deficient in ligase activity (Figures 1C and 2A) (Andrews *et al.*, 2005; Branzei *et al.*, 2006). *mms21-CH* mutants are highly sensitive to genotoxic agents and accumulate toxic DNA intermediates due to the failure to efficiently dissolve joint DNA molecules arising from homologous recombination or template switching (Andrews *et al.*, 2005; Branzei *et al.*, 2006; Sollier *et al.*, 2009).

We integrated *MMS21, mms21Δ18*, *mms21Δ22*, or *mms21-CH* alleles at the endogenous locus along with the *LEU2* selectable marker. Wild-type and mutant proteins generated by this method contain a 3HA epitope tag at the C-terminal end of the protein. *mms21-CH* mutants did not have any appreciable growth defect compared with the wild-type but were sensitive to MMS as reported in the literature (Andrews *et al.*, 2005; Branzei *et al.*, 2006; Sollier *et al.*, 2009). Unlike the ligase-deficient *mms21-CH* mutant, all eight clones of *mms21Δ22* mutants exhibited ∼10-fold growth defect compared with the wild-type (Figure 2A and B; Supplemental Figure S1, A and B). When treated with MMS, *mms21Δ22* mutants showed ∼1000-fold growth defect compared with the wild-type, suggesting that the growth defect was further exacerbated by MMS sensitivity (Figure 2B; Supplemental Figure S1, A and B). Intriguingly, growth defect and DNA damage sensitivity were more pronounced in *mms21Δ22* mutants than in ligase-defective *mms21-CH* mutants (Figure 2B). Contrary to *mms21Δ22* mutants, *mms21Δ18* cells did not exhibit any appreciable growth defect or DNA damage sensitivity, in agreement with structural predictions (Figures 1D and 2B). Because truncation of the last 22 amino acids, but not 18 amino acids, led to severe phenotypes, we designated the *mms21Δ22* mutation as the disease allele analogous to the pathogenic mutation found in human patients and the *mms21Δ18* mutation as the benign truncation (Figure 2A). Henceforth, we referred to the last 22 amino acids of *S. cerevisiae* Mms21 as the “C-terminus” for the rest of the study.

The C-terminus of Mms21 in *S. cerevisiae* contains a SUMO-interacting motif (SIM) (“AIDVL”) at the end of the protein (Figure 1A and C). This SIM, reported as SIM2 in the study by Varejão *et al.*, is involved in stabilizing the free SUMO non-covalently bound to Ubc9 from the backside and enhances the ligase activity of Mms21 (Varejao *et al.*, 2021). Consistent with the study by Varejão *et al.*, mutants that did not contain SIM2, *mms21sl* (sl = SIMless), grew comparable with the wild-type both in the absence and presence of MMS (Figure 2A and C) (Varejao *et al.*, 2021). Thus, the phenotypes of *mms21Δ22* could not be attributed to the lack of SIM2.

We confirmed the effects of the disease mutant analog, *mms21Δ22,* in a different system in which we generated the strains using the integration plasmid (Supplemental Figure S1C). In this system, wild-type and mutant Mms21 proteins were not HA-tagged. In the system with the integration plasmid, *mms21Δ22* mutants, but not *mms21-CH*, *mms21Δ18*, or *mms21sl* mutants, were susceptible to reversion even in the presence of the selection pressure (growth in the absence of leucine), further corroborating the idea that the disease allele analog disadvantaged cell growth (Supplemental Figure S1, C−F). Consistently, *mms21Δ22* mutants without reversion (cl. 3 in Supplemental Figure S1D) exhibited high sensitivity to MMS, whereas cells with the benign truncation (*mms21Δ18*) or without SIM2 (*mms21sl*) grew comparable with wild-type (Supplemental Figure S1F). Detailed descriptions of mutants with the integration plasmid system can be found in the Supplementary Materials. We observed that wild-type cells without the 3HA tag exhibited similar growth and DNA damage sensitivity compared with those containing the 3HA tag, suggesting that the 3HA tag did not significantly interfere with the function of the protein (Supplemental Figure S1G).

In addition to MMS, we tested whether the disease allele analog *mms21Δ22* influenced cells’ ability to resist other genotoxic agents. Similar to phenotypes observed in experiments with MMS, *mms21Δ22* mutants were highly sensitive to hydroxyurea, camptothecin, and ultraviolet radiation, but *mms21Δ18* and *mms21sl* mutants grew comparably with the wild-type (Figure 2, D–F). Taken together, we established that the C-terminus (last 22 amino acids) of *S. cerevisiae* Mms21 was crucial for the genome stability functions of the protein.

### Although the C-terminal truncation of Mms21 reduces its protein level, the phenotypes of *mms21Δ22* mutants cannot be attributed to diminished protein expression

Since truncation of the C-terminus might affect the stability of Mms21, we compared protein levels of *mms21Δ18, mms21Δ22*, and *mms21sl* mutants with the level of wild-type (Figure 2, G and H). Truncating the last 18 amino acids reduced the protein level by ∼50%, whereas loss of 22 amino acids diminished the protein level by ∼80% (Figure 2, G and H). Lack of SIM2 did not reduce the amount of Mms21 (Figure 2, G and H).

**FIGURE 3:**
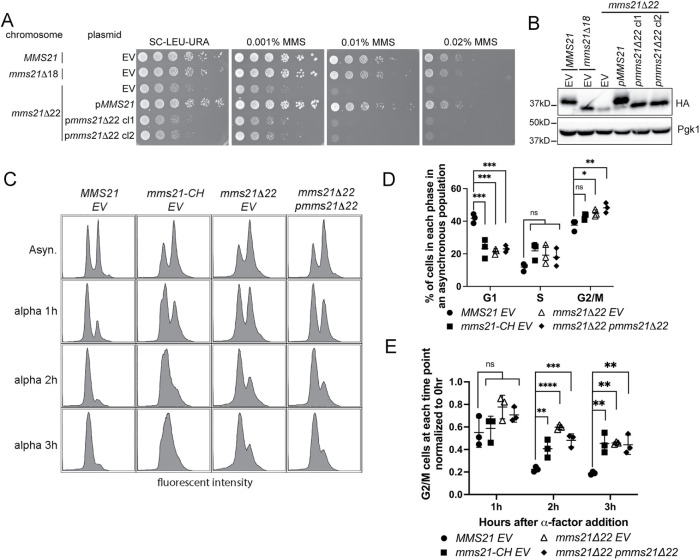
Restoring the level of the Mms21 mutant protein to the wild-type expression does not rescue *mms21Δ22* mutants’ phenotypes. **(A and B)** The *mms21Δ22* allele was overexpressed from pRS426 plasmid to restore the level of the protein. Controls include wild-type and *mms21Δ18* strains with empty pRS426. (EV = empty vector, p = plasmid). (**A)** Successive 10-fold serial dilutions of the indicated strains were grown on SC-LEU-URA without or with different concentrations of MMS (*n* = 2 for each clone, additional clones in Supplemental Figure S2). (**B)** Western blot analysis of the indicated strains in which both chromosomal and episomal copies of Mms21 wild-type or mutant proteins were epitope tagged with 3HA at the C-terminus and detected with an HA antibody. Pgk1 was used as a loading control. (cl. = clones). (**C)** Flow cytometry analysis of *MMS21*, *mms21-CH*, *mms21Δ22*, and *mms21Δ22*-overexpressing the *mms21Δ22* allele. *BAR1* was knocked out in all strains. The first row (Asyn.) represents an asynchronous population of each strain. When all strains reached mid-log phase, alpha factor (300 ng/ml) was added to the culture, and samples were collected 1 (alpha 1 h), 2 (alpha 2 h), and 3 (alpha 3 h) hours after the addition. DNA was stained with Sytox green. (**D)** The percentage of cells in each phase of the cell cycle in an asynchronous population was calculated from three experiments. Error bars represent SDs. (**E)** Relative amount of G_2_–M cells at each timepoint normalized to 0 h (at the time of alpha factor addition) calculated from three independent experiments. Error bars represent SDs. For both D and E, one-way ANOVA was performed to determine the statistical significance (**0.001, ***0.0002, ****<0.0001). (EV = empty vector, p = plasmid)

Reduced Mms21 protein stability in *mms21Δ22* mutants prompted us to investigate whether the phenotypes of these mutants with the disease allele analog were only due to a change in the amount of protein. To address this question, we overexpressed the *mms21Δ22* allele using pRS426, a high-copy plasmid (2µ circle plasmid), which generally yields 10 to 30 copies per haploid genome (Christianson *et al.*, 1992) (Figure 3A). The pRS426 plasmid containing the *mms21Δ22* allele was transformed into cells that also harbored the *mms21Δ22* mutation at the endogenous locus. Both episomal and chromosomal copies of the *mms21Δ22* allele contained DNA sequences encoding for 3HA tags at the C-terminus. As a control, we overexpressed the wild-type allele from pRS426 in the same *mms21Δ22* genetic background. Additional controls included wild-type and *mms21Δ18* (benign truncation) mutants with pRS426 empty vectors (Figure 3A). In these strains, the *MMS21* or *mms21Δ18* allele was expressed only from the endogenous locus.

We isolated four clones of *mms21Δ22* mutants with overexpression of *mms21Δ22,* and all of them exhibited Mms21 protein levels comparable with the level of wild-type expressed from the endogenous locus (Figure 3A and B; Supplemental Figure S2, A–C). Overexpression of the wild-type *MMS21* gene rescued the phenotypes of mutants with the disease allele analog, confirming that observed phenotypes were due to the mutation in the *MMS21* gene but not due to other secondary mutations (Figure 3A and B; Supplemental Figure S2, A–C). Although the episomal copy of *mms21Δ22* restored the level of the mutant protein to wild-type levels, it did not rescue the growth defect or MMS sensitivity of *mms21Δ22* mutants (Figure 3A and B; Supplemental Figure S2, A–C). These data imply that the C-terminal truncation to mimic the disease allele disrupted the functions of Mms21 by perturbing the structure, rather than altering protein levels.

### *mms21Δ22* mutants remain in the G_2_–M phase longer than wild-type cells

To gain insight into the growth defect of *mms21Δ22* mutants with the disease allele analog under unchallenged conditions, we compared the cell-cycle progression of these mutants with the wild-type and ligase-defective *mms21-CH* mutants. The asynchronous population of both *mms21Δ22* and *mms21-CH* mutants contained more G_2_–M phase cells compared with the wild-type population (although the difference between the wild-type and *mms21-CH* was not statistically significant) (Figure 3, C and D). Overexpression of the *mms21Δ22* allele did not significantly alter the cell-cycle profile of *mms21Δ22* mutants (Figure 3, C and D).

To closely monitor whether *mms21Δ22* mutants were delayed in G_2_–M, we added α-factor to arrest cells in G_1_ phase and determined the amount of G_2_–M cells at 1, 2, and 3 h after addition of α-factor. This experiment was performed in cells with *BAR1* knockout. The percentage of cells in G_2_–M at a given timepoint was determined relative to the percentage of G_2_–M cells in the asynchronous population. After 3 h in α-factor, ∼19% of the initial population of wild-type cells were in G_2_–M phase, whereas 44 to 45% of the initial population of *mms21-CH* and *mms21Δ22* cells remained in G_2_–M (Figure 3, C and E). Although *mms21-CH* mutants appeared to have a higher percentage of S-phase population than *mms21Δ22* mutants at 1- and 3-h timepoints, we could not detect quantitative differences (Figure 3C). A more sensitive readout is needed to discern whether there is any difference in S-phase timing between these two mutants.

### C-terminal truncation impairs ligase-dependent and -independent genome integrity functions of Mms21

Based on the phenotypes observed, we considered three possible scenarios. In the first scenario, the C-terminus contributed exclusively to the pathway dependent on the ligase activity. In the second scenario, the C-terminus functioned exclusively in a ligase-independent manner. In the third scenario, the C-terminus contributed to both ligase-dependent and -independent functions.

**FIGURE 4:**
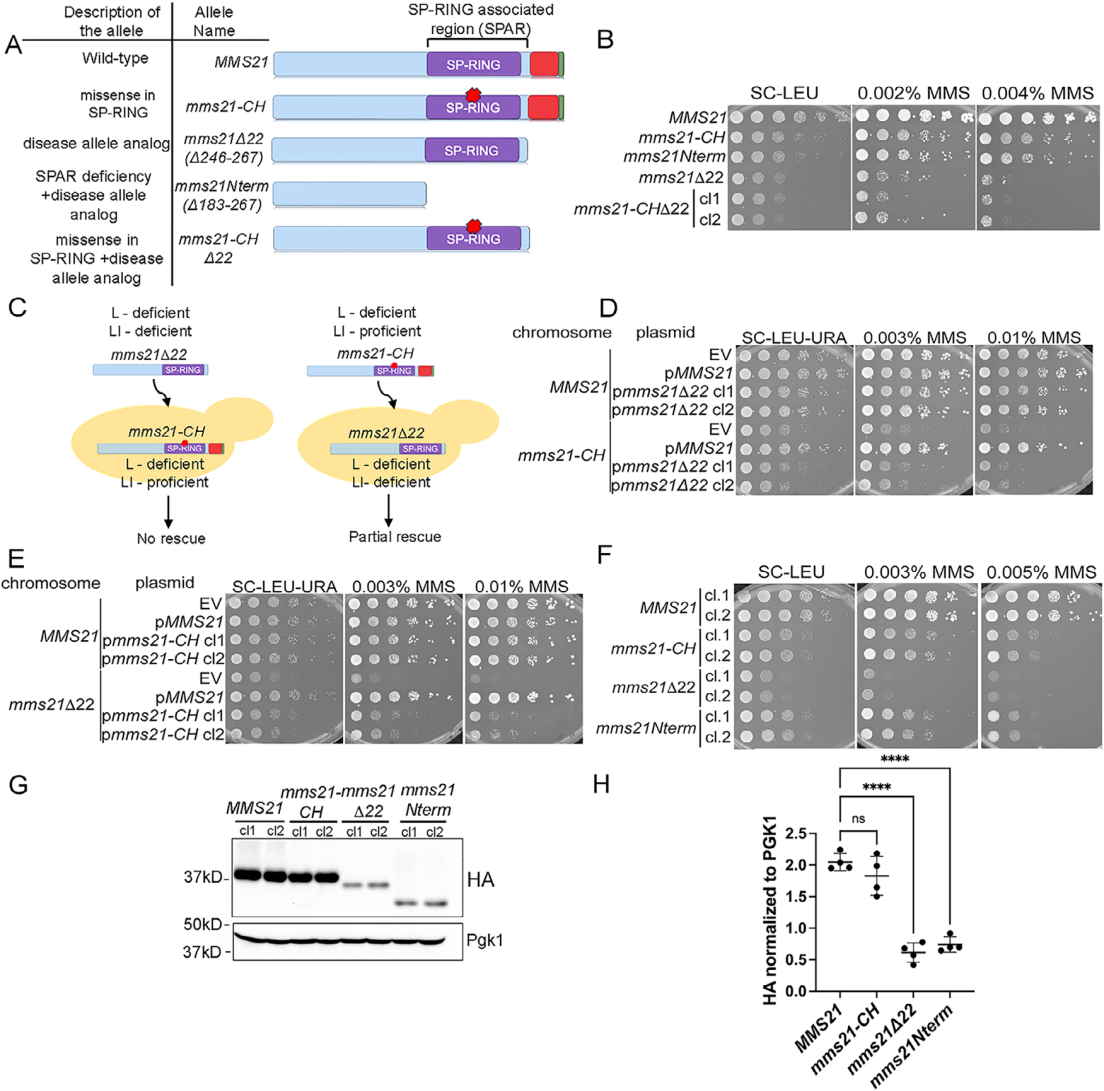
Genetic evidence demonstrating that the C-terminus contributes to ligase-dependent and -independent functions of Mms21 and restricts the function of the SPAR region to promote genome stability. **(A)** Different mutants of Mms21 used in Figure 4, and a description of each allele. (**B)** Successive 10-fold serial dilutions of the indicated strains were grown on SC-LEU without or with different concentrations of MMS (*n* = 3 for each clone). (**C)** Figure to illustrate the logic behind the rescue experiments in D and E. The *mms21Δ22* mutant, deficient in both ligase-dependent and -independent functions, cannot rescue the ligase-defective *mms21-CH* mutants. The *mms21-CH* mutant, proficient in the ligase-independent function but deficient in ligase activity, can partially rescue the DNA damage sensitivity of *mms21Δ22* mutants. (L = ligase, LI = ligase-independent function). (**D)** Successive 10-fold serial dilutions of the indicated strains were grown on SC-LEU-URA without or with different concentrations of MMS (*n* = 2 for each clone). Indicated alleles were expressed from pRS426 plasmids in *MMS21* or *mms21-CH* backgrounds. (**E)** Successive 10-fold serial dilutions of the indicated strains were grown on SC-LEU-URA without or with different concentrations of MMS (*n* = 2 for each clone). Indicated alleles were expressed from pRS426 plasmids in *MMS21* or *mms21Δ22* backgrounds. (EV = empty vector, p = plasmid). (**F)** Successive 10-fold serial dilutions of the indicated strains were grown on SC-LEU without or with different concentrations of MMS (*n* = 2 for each clone). (**G)** Western blot analysis of wild-type or mutant Mms21 proteins tagged with 3HA tags at the C-terminus. Pgk1 was used as a loading control. (**H)** Quantification of Western blots (*n* = 4). Error bars represent SDs. One-way ANOVA was performed to determine the statistical significance (****<0.0001) (cl. = clones).

To explore the first scenario, we compared *mms21Δ22* mutants with *mms21-CH* and *mms21Nterm* mutants—both of which have been known to be defective in the ligase activity. As stated above, *mms21-CH* mutants have two missense mutations in the SP-RING domain (Figures 1C and 4A). The *mms21Nterm* mutant is functionally equivalent to the well-characterized *mms21-11* allele—both lack the SP-RING domain and the C-terminus (missing amino acids 183-267) (Figures 1A and C, and 4A) (Zhao and Blobel, 2005). We use a different designation of the allele since *mms21-11* mutants were generated via transposon mutagenesis, whereas we generated *mms21Nterm* mutants via PCR-mediated integration (Zhao and Blobel, 2005). *mms21-11* mutants are deficient in ligase activity and are highly sensitive to DNA-damaging agents such as MMS and UV (e.g., Zhao and Blobel, 2005; Chen *et al.*, 2009). More pronounced phenotypes of *mms21Δ22* mutants with the C-terminal truncation compared with both ligase-deficient mutants excluded the first scenario (Figure 4A and B).

To test the second scenario, we generated *mms21-CHΔ22* mutants in which the ligase function was inactivated by two missense mutations in the SP-RING domain and the C-terminus was truncated (Figure 4A and B). If the C-terminus’ functions were exclusively ligase-independent, combining the ligase mutant and the C-terminal truncation should result in a worse phenotype than having an individual mutation. *mms21-CHΔ22* mutants phenocopied *mms21Δ22* mutants, suggesting that the second scenario is unlikely (Figure 4B). A simple explanation for this phenotype is that the C-terminal truncation already compromised the ligase activity and thus, two missense mutations in the SP-RING ligase domain did not exacerbate the lack of the C-terminus.

These data led us to the third scenario—the C-terminus functioned in both ligase-dependent and -independent manners. To validate this idea, we performed two rescue experiments. In the first experiment, we reasoned that, if the C-terminus positively regulated the ligase activity, the mutant protein without this region could not rescue the DNA-damage sensitivity of ligase-deficient *mms21-CH* mutants (Figure 4C, left panel). Our data supported this prediction that *mms21-CH* mutants with *mms21Δ22* overexpression exhibited similar DNA sensitivity as those without the overexpression, suggesting that the C-terminus positively contributed to the ligase activity (Figure 4D). In the second experiment, we hypothesized that, if an additional function of the C-terminus in genome integrity was independent of the ligase activity, the intact C-terminus in the ligase-deficient *mms21-CH* mutant should compensate for the lack of ligase-independent function in *mms21Δ22* mutants (Figure 4C, right panel). As a result, the *mms21-CH* mutant with the intact C-terminus should partially alleviate the phenotypes of *mms21Δ22* mutants (Figure 4C, right panel). Indeed, overexpressing the *mms21-CH* mutant in the *mms21Δ22* genetic background partially improved MMS sensitivity of *mms21Δ22* mutants (Figure 4E). Taken together, our genetic data suggested that the C-terminus of Mms21 could support both ligase-dependent and -independent genome integrity functions.

Comparison between different *mms21* mutants provided us with insights into the ligase-independent function of the C-terminus. Our data revealed that both *mms21Nterm* and *mms21-CHΔ22* mutants lacked the enzymatic function and the C-terminus (Figure 4A). Nevertheless, *mms21Nterm* mutants grew better than *mms21Δ22* mutants in the absence or presence of MMS, whereas *mms21-CHΔ22* phenocopied those cells with the disease allele analog (Figure 4, B and F). The *mms21Nterm* mutant has a larger truncation compared with the *mms21Δ22* mutant, and the protein level is similar to that of the *mms21Δ22* mutant, further confirming that the protein level does not explain the phenotypes of *mms21Δ22* mutants (Figure 4A, G, and H). Instead, these data suggested that the function of the C-terminus was crucial when the nonenzymatic activity of the SP-RING–associated region (SPAR) (amino acid 183-245) was present (Figure 4A).

One possibility is that the C-terminus regulates the SPAR region, which would otherwise interfere with homeostasis of a genome stability pathway. As a result, when the SPAR was deleted in the *mms21Nterm* mutant, the absence of the C-terminus was tolerated (Figure 4A, B, and F). The SPAR region contains Ubc9 binding sites, and thus, we entertained the possibility that the C-terminus promoted the release of Ubc9 from its association with the SPAR region (Duan *et al.*, 2009). As a result, Ubc9 might be sequestered by the *mms21Δ22* protein due to the lack of the C-terminus, limiting the ability of Ubc9 to interact with other E3 SUMO ligases. If this prediction could explain the phenotype, overexpression of Ubc9 should alleviate the phenotypes of *mms21Δ22* mutants. However, we found that overexpressing Ubc9 did not rescue the lack of the C-terminus (Supplemental Figure S3, A–C).

## DISCUSSION

Based on its diverse roles in genome integrity, the Smc5/6/Mms21 complex is considered to be a “guardian of the genome” (Peng and Zhao, 2023). The role of Mms21 in genome integrity is further underscored by the highly conserved nature of this protein and its obligate partner, the Smc5/6 complex, in multiple organisms such as yeast, human, and mice (Peng and Zhao, 2023). To date, our understanding of Mms21’s functions in genome integrity is mostly based on ligase-deficient mutants of Mms21. Mms21’s functions beyond the ligase activity have not been well characterized, partly due to the lack of other hypomorphic mutants. In this study, we successfully characterized a hypomorphic mutant of *S. cerevisiae MMS21*, analogous to a human disease allele of *NSMCE2*, to better understand the functions of Mms21 in genome stability. Using this disease allele analog, we were able to connect ligase-dependent and -independent functions of Mms21 to the C-terminus of the protein. Our genetic data provided evidence that the C-terminus cooperated with the SPAR region of the protein to optimize genome stability conditions.

### The C-terminus of Mms21 contributes to ligase-dependent and -independent functions

Different lines of evidence suggest that the lack of the C-terminus impairs Mms21’s ligase activity. First, *mms21Δ22* mutants exhibited a G_2_–M cell cycle delay similar to *mms21-CH* mutants (Figure 3, C–E). Second, *mms21-CHΔ22* mutants phenocopied *mms21Δ22* mutants, suggesting that the C-terminally truncated mutant was already deficient in the ligase activity and could not be further exacerbated by mutations in the ligase domain (Figure 4A and B). Third, overexpressing the *mms21Δ22* allele did not rescue the DNA damage sensitivity of ligase-deficient *mms21-CH* mutants (Figure 4D). Our data set the stage to determine whether the disease allele analog *mms21Δ22* undermines the ligase activity in a direct or indirect manner.

Evidence from the literature suggests that having an intact SP-RING domain is not sufficient, but additional regulations are needed for the optimal ligase activity of Mms21. The C-terminus may contribute to one of these regulatory pathways. For instance, ATPase activity and DNA binding of Smc5 enhance the SUMO ligase activity of Mms21 (Bermudez-Lopez *et al.*, 2015; Varejao *et al.*, 2018). The crystal structure of Mms21 bound to Smc5 reveals that the four amino acids (“KIAK”) present in the benign truncation (*mms21Δ18)*, but not present in the disease allele analog (*mms21Δ22*), are in proximity to the N-terminus (Figure 1D). The N-terminus of Mms21 interacts with Smc5 (Duan *et al.*, 2009; Peng and Zhao, 2023). Thus, it is possible that the lack of KIAK residues in the disease allele analog destabilizes the Mms21–Smc5 interaction and impairs the ligase activity of Mms21. Another intriguing possibility is that the C-terminus regulation of the E3 ligase activity is crucial only in the context of chromatin-bound Smc5/6 complex when coordination among multiple regulatory pathways is necessary to tightly control the enzymatic activity of Mms21 inside the cell. In this model, the C-terminus may promote the function of the catalytically active SP-RING by scaffolding selective substrates. In an alternative scenario, the lack of KIAK residues close to the SP-RING domain may be one contributing factor that changes the conformation of the ligase domain and directly impair the catalytic activity of the enzyme (Figure 1D). Our conclusion about the contribution of the C-terminus to the ligase activity is mainly based on genetic evidence—we did not examine sumoylation of known substrates of Mms21, such as Smc5, in *mms21Δ22* mutants. In human cells, the C-terminal truncation does not appear to significantly reduce the ligase activity of the protein (Payne *et al.*, 2014). It is noteworthy that the ligase activity of the human p.Ala234Glufs*4 Mms21 mutant was examined in a system in which both SUMO and the mutant Mms21 were overexpressed while the endogenous Mms21 was still present (Payne *et al.*, 2014). Further studies are necessary in both yeast and mammalian cells to better elucidate the role of the C-terminus in Mms21’s ligase activity.

**FIGURE 5:**
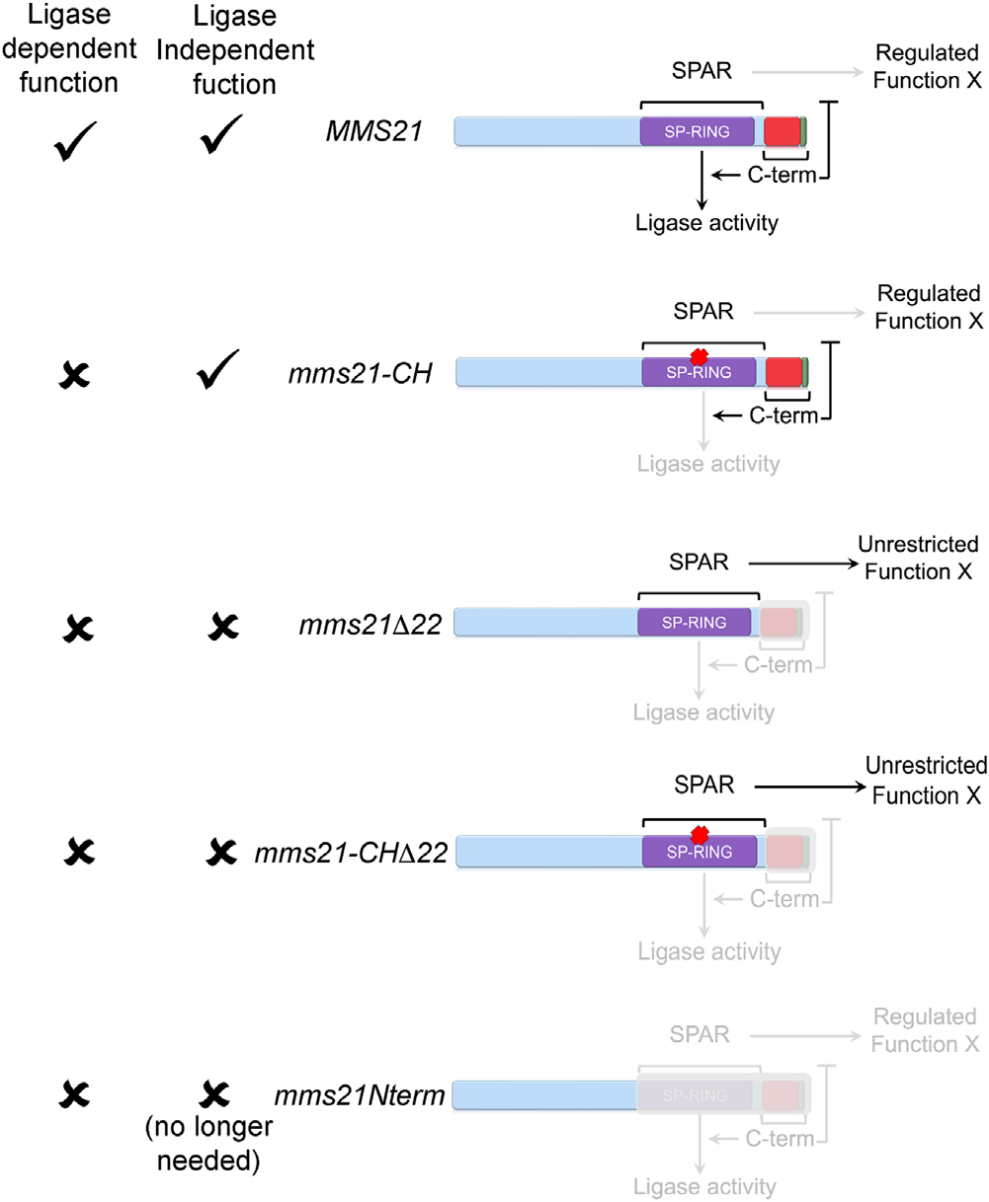
Proposed model of how different regions of Mms21 mediate ligase-dependent and -independent functions. Both ligase-dependent and -independent functions are crucial to achieve optimal genome stability. In *mms21-CH*, the ligase activity is deficient, but the functional C-terminus regulates the activity SPAR. In *mms21Δ22* and *mms21-CHΔ22* mutants, both ligase-dependent and -independent functions are deficient. Unregulated SPAR activity in this condition interferes with genome stability. In *mms21Nterm* mutants, the SPAR region is absent. As a result, the ligase-independent function of the C-terminus is no longer needed to restrain SPAR.

Our data further suggest that the C-terminus regulates other functions beyond the ligase activity. This is most evident by more pronounced growth defects and increased DNA damage sensitivity of *mms21Δ22* mutants compared with *mms21-CH* mutants (Figures 2B and 4, B and F). In agreement with this observation, overexpressing the *mms21-CH* allele could partially rescue the defects of *mms21Δ22* cells (Figure 4E). This partial rescue suggested that the intact C-terminus of the mutant Mms21 protein with two missense mutations in the ligase domain could carry out the ligase-independent genome stability function (Figures 4, C and E, and 5). Based on our genetic data, we proposed that the ligase-independent function of the C-terminus was to modulate the activity of the SPAR region, as discussed below.

Our findings are in agreement with the SUMO ligase-independent function(s) of Mms21 reported in mice. Transgenic mice with two-point mutations that disabled the ligase activity did not exhibit aging phenotypes, but conditionally knocking out *NSMCE2* in adult mice led to premature aging and characteristics reminiscent of Bloom's syndrome (Jacome *et al.*, 2015). These phenotypes may be attributed to the N-terminus of Mms21 in facilitating its interaction with Smc5 and Smc6, as suggested by the study in *S. cerevisiae* (Duan *et al.*, 2009; Li *et al.*, 2024). Smc5–Mms21 interaction via the N-terminus of Mms21 is thought to be essential for cellular survival (Duan *et al.*, 2009). In addition, it is also possible that ligase-independent function(s) of the C-terminus contributes to the phenotypes observed in conditional *NSMCE2* knockout mice. SUMO ligase-independent functions of Mms21 in chromosome segregation during meiosis have also been recognized in *S. cerevisiae* (Xaver *et al.*, 2013). In meiosis, the Smc5/6/Mms21 complex has a role in promoting resolvases, but this function does not appear to depend on the ligase domain (Xaver *et al.*, 2013).

Our data exclude the possibility that the effects on both ligase-dependent and -independent functions are simply due to low levels of Mms21. Restoring the expression of the *mms21Δ22* allele to the wild-type level did not rescue the phenotypes (Figure 3A and B; Supplemental Figure S2, A–C). Furthermore, the levels of Mms21 in *mms21Nterm* and *mms21Δ22* mutants were similar, and yet the former mutant exhibited better resistance to genotoxic stress than the latter, further emphasizing that protein levels merely did not account for the phenotypes of *mms21Δ22* mutants (Figure 4, F–H).

### The C-terminus of Mms21 supports genome stability during normal replication

The poor growth phenotype of *mms21Δ22* mutants with the disease allele analog suggests that the C-terminus ensures genome stability even under unperturbed growth conditions. Notably, missense mutations disabling the ligase activity of Mms21 in *mms21-CH* mutants did not significantly impede growth under unperturbed conditions compared with *mms21Δ22* mutants (Figures 2A and B and 4, B and F). The noticeable growth defect of *mms21Δ22* mutants is reminiscent of the marked slow growth of cells in which Mms21 expression was restricted to the S-phase (Menolfi *et al.*, 2015). Restriction of Mms21 expression to the S-phase is associated with increased chromosome breakage, and this phenotype can be explained by the function of the Smc5/6 complex and Mms21 in processing structures that arise from DNA damage tolerance pathways to alleviate endogenous replication stress (Menolfi *et al.*, 2015). These functions are needed during normal replication to overcome difficult-to-replicate regions within the genome. Thus, it is conceivable that the C-terminus alleviates endogenous replication stress together with or independent of the ligase function. Consistent with this notion, patient cells with *NSMCE2* compound heterozygous mutants exhibited a high level of micronuclei formation even under unchallenged conditions, reinforcing the idea that the C-terminus is crucial for resisting endogenous genome instability (Payne *et al.*, 2014).

Failure of the disease allele analog to counteract endogenous genome instability explains the G_2_–M delay of *mms21Δ22* mutants. Insufficient ability to process aberrant structures during normal replication can delay mitosis. For instance, in *dna2* helicase mutants, stalled replication forks could not be processed efficiently, leading to mitotic delay (Olmezer *et al.*, 2016). Likewise, mild replication stress, either induced by a partial defect in a replication protein or a low dose of MMS, subsequently led to mitotic delay (Thu *et al.*, 2016). In mammalian cells, the mitotic checkpoint has been shown to respond to transient replication stress (Duensing *et al.*, 2006). G_2_–M delay of *mms21Δ22* mutants is consistent with data from human cells. In human cells with compound heterozygous mutations in *NSMCE2*, an increase in mitotic population was also observed (Deiss *et al.*, 2019). Similarly, *NSMCE2* deletion in mice thymus cells resulted in mitotic cell accumulation (Jacome *et al.*, 2015). It is important to note that the cell-cycle delay is also observed in *mms21-CH* mutants, suggesting that the cell-cycle delay phenotype does not fully account for the slow growth of *mms21Δ22* mutants.

### The C-terminus counteracts the function of the adjacent SPAR region in a ligase-independent manner to support genome stability

The Smc5/6 complex and Mms21 contribute to different steps of regulating recombination structures. Mms21 regulates processes that can generate recombinogenic structures. For instance, sumoylation of Mph1 helicase by Mms21 facilitates the helicase's ability to generate regressed forks, which may be converted to aberrant recombination structures (Meng and Zhao, 2017; Zapatka *et al.*, 2019). Similarly, the pro-recombinogenic function of Mph1 is toxic in *mms21-11* mutants, suggesting that Mms21 restrains the activity of Mph1 (Chen *et al.*, 2009). In addition, Mms21 is crucial for limiting recombinogenic structures. Mms21, as part of the Smc5/6 complex, has been implicated in dissolution and resolution pathways, both of which process DNA structures that arise from recombination (Xaver *et al.*, 2013; Bermudez-Lopez *et al.*, 2016; Bonner *et al.*, 2016; Pond *et al.*, 2019).

The intriguing possibility is that different regions of Mms21 independently or synergistically function to regulate diverse roles of Mms21 in the generation or clearance of recombinogenic structures. Our genetic data suggest that the activity of the C-terminus is crucial only in the presence of the SPAR region and that the C-terminus counteracts the function mediated by the SPAR region (Figure 5). The phenotypic differences between *mms21Δ22* and *mms21Nterm* mutants supported these ideas. Removing the SPAR region rescued the negative phenotypes associated with the C-terminal deficiency, suggesting that the C-terminus restrained the function of the SPAR, which would otherwise interfere with processes promoting genome stability (Figures 4A, B, and F, and 5). Furthermore, our genetic data implied that the interfering activity of the SPAR did not depend on the ligase activity. The phenotypic similarities between *mms21Δ22* and *mms21-CHΔ22* mutants validated this idea—negative phenotypes associated with the unregulated SPAR region persisted when the SP-RING domain was mutated (Figures 4B and 5). Finally, our genetic evidence suggested that the ligase-independent function of the C-terminus is to tightly control the function of the SPAR region. Improved growth and DNA damage sensitivity of *mms21-CH* mutants compared with *mms21Δ22* mutants and the partial rescue of *mms21Δ22* cells by the *mms21-CH* mutant supported this conclusion—the intact C-terminus could restrain the SPAR independently of the ligase function (Figures 2B, 4, E and F, and 5).

We explored one possibility that might reveal the mechanisms by which the C-terminus relieved the interfering effects of the SPAR. The SPAR contains Ubc9-binding sites and lack of the C-terminus may prolong association of Ubc9 with Mms21 (Duan *et al.*, 2009). In this model, the *mms21Δ22* mutant may act as a dominant negative mutant, preventing the Ubc9 E2 enzyme from working together with other E3 ligases, Siz1 and Siz2. However, our data suggest that negative effects through sequestration is unlikely. The endogenous expression level of the *mms21Δ22* mutant was significantly reduced to compete for Ubc9 against other proteins. Furthermore, when the *mms21Δ22* mutant was overexpressed in *MMS21* or *mms21-CH* background, cell growth and DNA damage sensitivity were comparable with cells without the overexpression (Figure 4D). These data implied that the mutant, at least in the context of these experimental conditions, did not behave as a dominant negative mutant. In line with this notion, attempting to overcome the potential limitation of Ubc9 availability by overexpressing Ubc9 in *mms21Δ22* mutants did not rescue the phenotypes of the mutants (Supplemental Figure S3, A–C). Although sequestering Ubc9 does not explain the phenotypes, it is reasonable to imagine that the SPAR serves as a scaffold for Mms21 to interact with another genome stability protein and that this interaction must be regulated by the C-terminus to optimize repair or tolerance mechanisms (Figure 5).

We acknowledge that the scope of our study is limited to genetic evidence, suggesting potential mechanisms by which Mms21’s C-terminus contributes to genome stability. Our study did not examine whether the lack of the C-terminus disrupts DNA replication or recombination, processes known to be regulated by Mms21 and the Smc5/6 complex (Peng and Zhao, 2023). G_2_–M delay phenotypes associated with the C-terminal truncation in *mms21Δ22* mutants imply that these cells may experience replication stress and subsequent chromosome segregation errors. Likewise, we did not examine the localization of the C-terminally truncated Mms21 mutant. Direct biochemical or cellular assays are necessary to better understand the contributions of the C-terminus to Mms21’s functions.

In this study, we successfully modeled the human disease allele of *NSMCE2* in *S. cerevisiae* using the yeast analogous allele. Through this genetic model, we were able to relate cellular phenotypes to the structure of the protein and infer ligase-dependent and -independent functions of Mms21. Insights from the C-terminal truncation of Mms21 in the *S. cerevisiae* genetic model can potentially explain genome instability phenotypes associated with compound heterozygous mutants of *NSMCE2* in a rare human genetic condition (Payne *et al.*, 2014). Furthermore, the *mms21Δ22* mutant will be a valuable hypomorphic allele for future genetic interaction studies to reveal further mechanistic insights into the etiology of the genetic condition, as well as the principles of how an E3 SUMO ligase orchestrates genome integrity pathways.

## MATERIALS AND METHODS

Request a protocol through *Bio-protocol*

### Yeast strains and primers

Yeast strains used in this study are isogenic derivatives of BY4741. The BY4741 parental strain was purchased from Horizon Discovery. A complete list of strains used in this study can be found in Supplemental Table S1 (Supplementary Material). Primers used for cloning and strain construction are in Supplemental Table S2 (Supplementary Material).

### Construction of *mms21* mutants using the integration vector-dependent system

The 3′ fragment of *MMS21* wild-type or *mms21* mutant genes (from 321 to the STOP codon) were cloned into pRS405 by HiFi Assembly (NEB E2621). All PCR reactions were performed using Phusion Master Mix (NEB M0531S). The gene fragments were generated by PCR using genomic DNA of BY4741 (or a strain with *mms21-CH* (C200/202H) mutations) as template and the following primers: mms21_F and one of the reverse primers depending on which mutant of *MMS21* was generated: mms21_R, mms21-d22_R, mms21_d18_R, or mms21-simless_R. The primers contain regions homologous to sequences in pRS405. pRS405 was linearized by digestion with HindIII-HF (NEB R3104) and BamHI-HF (NEB R3136). Ligated pRS405 plasmids were transformed into bacteria, and the resulting colonies were screened using 5pRS316 and 3pRS400 primers. Plasmids from positive colonies were verified by Sanger sequencing.

To integrate the gene at the endogenous locus, pRS405 with *MMS21* or *mms21* was linearized by NdeI-HF (NEB R3131S) and transformed into BY4741 strains. The integration was verified by using these primers: Mms21_50up and 3pRS400, and the PCR products were Sanger sequenced for verification.

To detect revertants, Mms21_50up and Mms21_56dn primers were used. These primers anneal to regions 50 base pairs upstream or 56 base pairs downstream of the *MMS21* endogenous gene. In cells that retained the transgene, these primers did not efficiently amplify a product since the size is ∼6000 base pairs.

### Construction of *mms21* mutants with the 3HA tag using the two-step PCR method

All PCR reactions were performed using Phusion Master Mix (NEB M0531S). The 3′ half of the *MMS21* gene without or with desired mutations and the 3HA tag were cloned into the pRS405 integration plasmid using double digest with restriction enzymes. PCR products were generated as described below. The first PCR product containing the 3′end of *MMS21* or *mms21* and sequences of the first HA tag was generated using genomic DNA of BY4741 as template and these primers: 5HindIII_mms21_321 and one of these primers depending on which mutant of *MMS21* was generated: 3MMS21-no stop-NheI-1HA, 3mms21d18-no stop-NheI-1HA, 3mms21d22-no stop-NheI-1HA, 3mms21sl-no stop-NheI-1HA, or 3mms21Nterm-no stop-NheI-1HA. For the *mms21-CH* mutant, genomic DNA of the *mms21-CH* strain was used as template along with these primers: 5HindIII_mms21_321 and 3MMS21-no stop-NheI-1HA. For the *mms21-CHΔ22* mutant, genomic DNA of the *mms21-CH* strain was used as template along with these primers: 5HindIII_mms21_321 and 3mms21d22-no stop-NheI-1HA. The second PCR product containing the 3′end of *MMS21* or *mms21* and sequences of two HA tags was generated using the first PCR product as template and these primers: 5HindIII_mms21_321 and 3_2HA. The third PCR product containing the 3′end of *MMS21* or *mms21* and sequences of three HA tags followed by a stop codon was generated using the second PCR product as template and these primers: 5HindIII_mms21_321 and 3_3HA-STOP-BamHI. The third PCR product was incorporated into pRS405 by double digest with HindIII-HF and BamHI-HF. Ligated pRS405 plasmids were transformed into bacteria, and the resulting colonies were screened using 5pRS316 and 3pRS400 primers. Plasmids from positive colonies were verified by Sanger sequencing. In *mms21-4A* with the 3HA (generated by Genewiz Mutagenesis service), KIAK (a/a 246-249) was mutated to AAAA.

To generate the first fragment for integration, pRS405 carrying *MMS21-3HA* or a *mms21* mutant with the 3HA tag was used as template along with these primers: 5Mms21_400 and 3pRS40x_3HA. To generate the second fragment for integration, pRS405 was used as a template along with these primers: 5pRS40x_2step and 40dnMms21_LEU2_r. The first fragment contains the 3′end of *MMS21* or *mms21* with DNA sequences of the 3HA tag and a region homologous to the second fragment. The second fragment contains a region homologous to the first fragment as well as the *LEU2* auxotrophic marker. The first and second fragments were transformed into the BY4741 strain in which *BAR1* was knocked out. Integration of the transgene was screened by PCR using the following primers: Mms21_50up and 3LEU2_880pRS405, and all PCR products were Sanger sequenced for verification.

### Knocking out *BAR1*

The *BAR1* gene was knocked out by using a PCR product that contained the *NatMX* gene flanked by homologous regions 40 base pairs upstream or downstream of the gene. PCR product was created by using pFA6a-natMX6 plasmid (EuroScarf p30437) as template and the following primers: Bar1KONat_f and Bar1KONat_r. Clones were selected on YPD plates containing 100 mg/ml nourseothricin (GoldBio N-500-100). Integration of the *NatMX* gene was confirmed by PCR using these primers: Bar1_120up and Bar1_166dn and PCR products were Sanger sequenced.

### Generation of cells overexpressing pRS426-*MMS21* or *mms21-*3HA

We cloned *MMS21-3HA* or *mms21-3HA* into pRS426. All PCR reactions were performed using Phusion Master Mix (NEB M0531S). To generate inserts, genomic DNA of *MMS21-3HA* or *mms21-3HA* mutants were used as template along with these primers: HindIII_yMMS21_f and MMS21_NotI_r. These inserts were cloned into pRS426, linearized with HindIII-HF and NotI-HF, by HiFi Assembly (NEB E2621). Success of insertion was confirmed by PCR using 5pRS316 and 3pRS400 primers and sequences were verified by Sanger sequencing.

pRS426-*MMS21-3HA*, *mms21-3HA* or pRS426 empty vector was transformed into cells with *MMS21-3HA* or *mms21-3HA* at the endogenous locus. Both episomal and chromosomal alleles were verified by PCR and by Sanger sequencing.

### Generation of cells overexpressing pRS423-*UBC9-Flag*

We cloned *UBC9-Flag* into pRS423. All PCR reactions were performed using Phusion Master Mix (NEB M0531S). The *UBC9* gene was cloned as described below. The first fragment containing the exon 2 of *UBC9* with sequences encoding for the Flag tag was generated using BY4741 genomic DNA as template and the following primers: 5Ubc9_ex2 and 3FlagUbc9_noSTOP. The second fragment containing both exons of *UBC9* and sequences encoding for the Flag tag was generated using the first fragment as template and the following primers: 5Ubc9_ex1 and 3STOP_Flag. The third fragment containing the *UBC9* gene with sequences encoding the Flag tag flanked by regions homologous to pRS423 was generated using the second fragment as template and the following primers: 5pRS423_EcoRI_Ubc9 and 3pRS423_BamHI_Flag. The third fragment was ligated into pRS423, linearized by EcoRI-HF and BamHI-HF, using HiFiAssembly (NEB E2621). Success of insertion was confirmed by PCR using 5pRS316 and 3pRS400 primers, and sequences were verified by Sanger sequencing.

pRS423-*UBC9-Flag* or pRS423 empty vector was transformed into cells that carry *MMS21-3HA* at the endogenous locus and pRS426 or cells that carry *mms21Δ22-3HA* at the endogenous locus and pRS426-*mms21Δ22-3HA*. Both episomal and chromosomal genotypes (*MMS21*, *mms21*, or *UBC9*) were verified by PCR and by Sanger sequencing.

### Preparation of whole-cell extract and Western blot analysis

Whole-cell extracts were prepared by trichloroacetic acid (TCA) precipitation. Briefly, cell pellets were washed once with 1 ml of 20% TCA, followed by lysis with acid-washed glass beads (425−600 µm, Sigma G8772) in 100 µl of 20% TCA. The samples with glass beads were vortexed in 4°C for 5 min using the bead beater. 1 ml of 20%TCA was added to each tube with beads to facilitate liquid transfer to a new Eppendorf tube. Then the samples were centrifuged at 3000 rpm for 10 min to precipitate proteins, followed by removal of the supernatant. To each precipitated pellet, 50 to 100 µl of 2x Lammeli buffer with 200 mM of dithiothreitol was added, during which the samples turned yellow. To adjust the pH, the minimum possible volume of 1 M Tris base (typically 35−40 µL) was added dropwise until a color change from yellow to blue was observed. Samples were then boiled for 5 min, followed by centrifugation at 3000 rpm for 10 min. Supernatant was transferred to a new set of tubes, and the insoluble portion was discarded. TCA samples were quantified using the RC DC Protein Assay Kit II (Bio-Rad 5000122).

For Western blot analysis, 25 µg of whole-cell extract for each sample was loaded onto the SDS–PAGE gel, followed by immunoblotting. For Mms21 wild-type and mutant proteins, 7% SDS–PAGE gel was used, and the proteins were detected using an HA antibody (1:1000 in 5% milk 1xPBS) (Roche 3F10). For Ubc9, 10% SDS–PAGE gel was used, and the Flag-tagged Ubc9 was detected using a Flag antibody (1:1000 in 5% milk 1xPBS) (Sigma M2). Pgk1, detected by an anti-Pgk1 antibody (1:2000 in 5% milk 1xPBS) (Abcam 22C5D8), was used as a loading control.

### Spotting assay

From liquid cultures grown overnight, a total of 2 × 10^7^ cells were transferred to an Eppendorf tube. Cells were pelleted by centrifugation, resuspended in 300 µL of sterile water, and transferred to the first column of a 96-well plate. Using this as the most concentrated sample, a serial 10-fold dilution was performed in each successive column of the plate. Cells were transferred to an agar plate with appropriate media using a multi-pronged spotting manifold, followed by growth for 2 to 5 d. Spotting was performed with at least two independently isolated clones.

### Flow cytometry

All strains were grown overnight in SC-LEU-URA. The next morning, they were diluted to OD_600_ = 0.1 and were grown for 3 h when they reached approximately OD_600_ = 0.3 to 0.4. Mid-log phase asynchronous cultures were collected and fixed with 70% ethanol. The Alpha factor (300 ng/ml) was added to the remaining culture, and samples were collected every hour for 3 h and fixed with 70% ethanol. The Alpha factor was replenished after 1.5 h.

Samples were processed according to the protocol by Rosebrock, 2017 (Rosebrock, 2017). Briefly, fixed cells were incubated in 800 µL of sodium citrate buffer (pH = 7.4) twice, followed by 1 to 2 h incubation with 500 µL of sodium citrate buffer containing 20 µg/ml RNase (Sigma R4642) and 2.5 µM of Sytox Green (Invitrogen S7020) at 37°C in the dark. After RNase treatment, samples were incubated with proteinase K (10 µL of 20 mg/ml stock for each sample, Thermo Fisher Scientific, EO0491) at 55°C for 1 to 2 h in the dark. Samples were stored in the dark in 4°C overnight. Samples were sonicated before subjected to flow cytometry using Cell Sorter SH800S (Sony Biotechnology).

Flow cytometry experiments were repeated three times (three biological replicates). The percentage of cells in each phase of the cell cycle in an asynchronous population and fold changes in G_2_–M cells at each timepoint normalized to 0 h (at the time of alpha factor addition) were calculated from three experiments. Using GraphPad Prism, one-way ANOVA was performed to determine the statistical significance (**0.001, ***0.0002, ****<0.0001).

## Supporting information





## Abbreviations used:

SPAR: SP-RING–associated region
STR complex: Sgs1, Top3, and Rmi1 complex.
