# Supplementary material for: Ligase-dependent and independent functions of the C-terminus of Mms21 contribute to optimal growth and genome stability in Saccharomyces cerevisiae
Source: Mol Biol Cell. 2026 Apr 8;37(6):ar50. doi: 10.1091/mbc.E25-11-0567 (PMC13200702; doi:10.1091/mbc.E25-11-0567)
Supplement: Supplementary file 1 [file mbc-37-ar50-s001.pdf]

Supplemental Materials

Molecular Biology of the Cell

Li *et al*.

Supplemental Figure 1

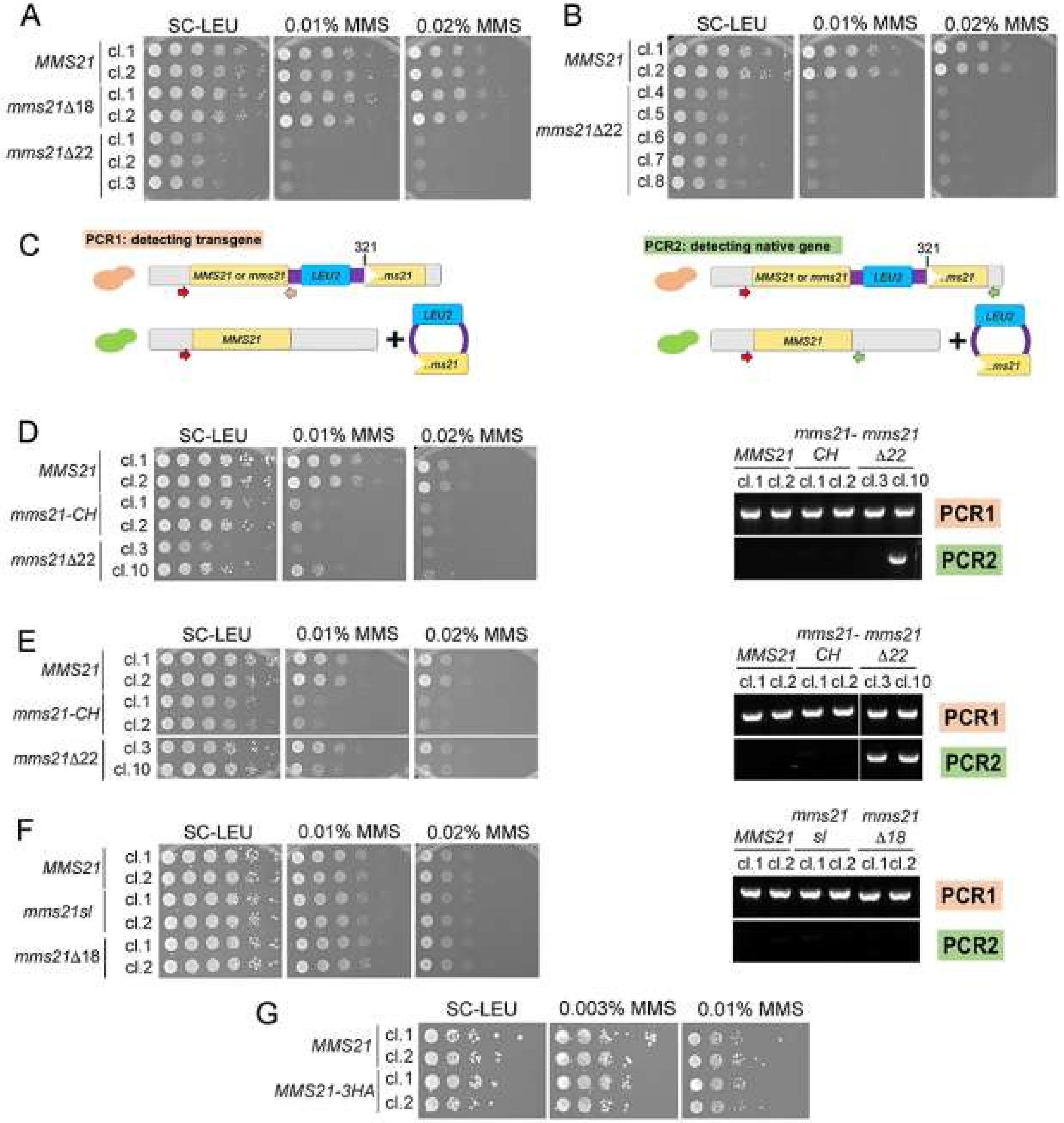

Supplemental Figure 1. Independent clones of *mms21Δ22* mutants show consistent growth defect and sensitivity to MMS. **A and B)** Successive 10-fold serial dilutions of indicated strains grown on SC-LEU without or with different concentrations of MMS (n = 2 for each clone). (cl. = clones) **C)** Diagrams illustrating two different pairs of primers that detect the transgene or the native gene in wild-type or *mms21* mutants. The primer pair that detects the native gene does not amplify the transgene with the *LEU2* marker since the product is ~6000bp. **D-G)** Successive 10-fold serial dilutions of indicated strains grown on SC-LEU plates without or with different

concentrations of MMS. The gel images illustrate the detection of the transgene (PCR1, top) or the native gene (PCR2, bottom). The experiment in **E)** was performed with strains from an earlier generation, whereas **F)** was performed with strains from a later generation. The line in F represents deletion of a lane not relevant for the figure. Each spotting could be performed only once due to reversion. We observed similar phenotypes associated with reversion in two additional independent *mms21Δ22* clones. **G)** Comparison between wild-type without or with 3HA tags. (n =1 for each clone) (cl. = clones)

Supplemental Figure 2

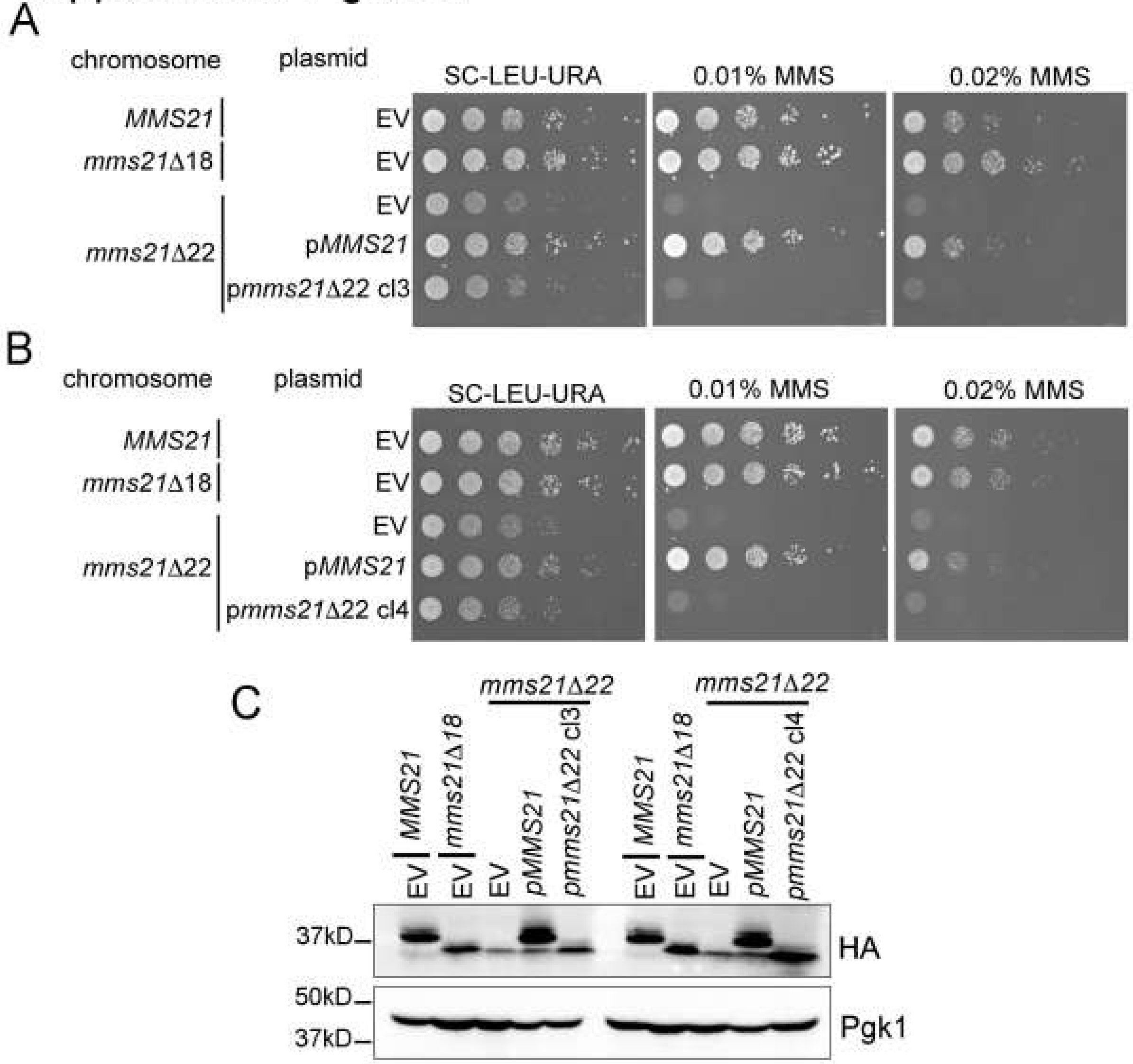

Supplemental Figure 2. Independent clones of *mms21Δ22* mutants overexpressing the *mms21Δ22* allele demonstrate that restoring the level of the mutant protein does not rescue the phenotypes of *mms21Δ22* mutants. **A-B)** Different clones of *mms21Δ22* mutants overexpressing the *mms21Δ22* allele from the pRS426 plasmid. *MMS21* and *mms21Δ18* strains with empty vector

were controls. Successive 10-fold serial dilutions of indicated strains grown on SC-LEU-URA without or with different concentrations of MMS (n = 2 for each clone). (EV = empty vector, p = plasmid). **C)** Western blot analysis of strains with C-terminally 3HA tagged Mms21 wild-type or mutant proteins. Both chromosomal and episomal copies of Mms21 were C-terminally 3HA tagged and detected with a HA antibody. Pgk1 was a loading control. (cl. = clones)

Supplemental Figure 3

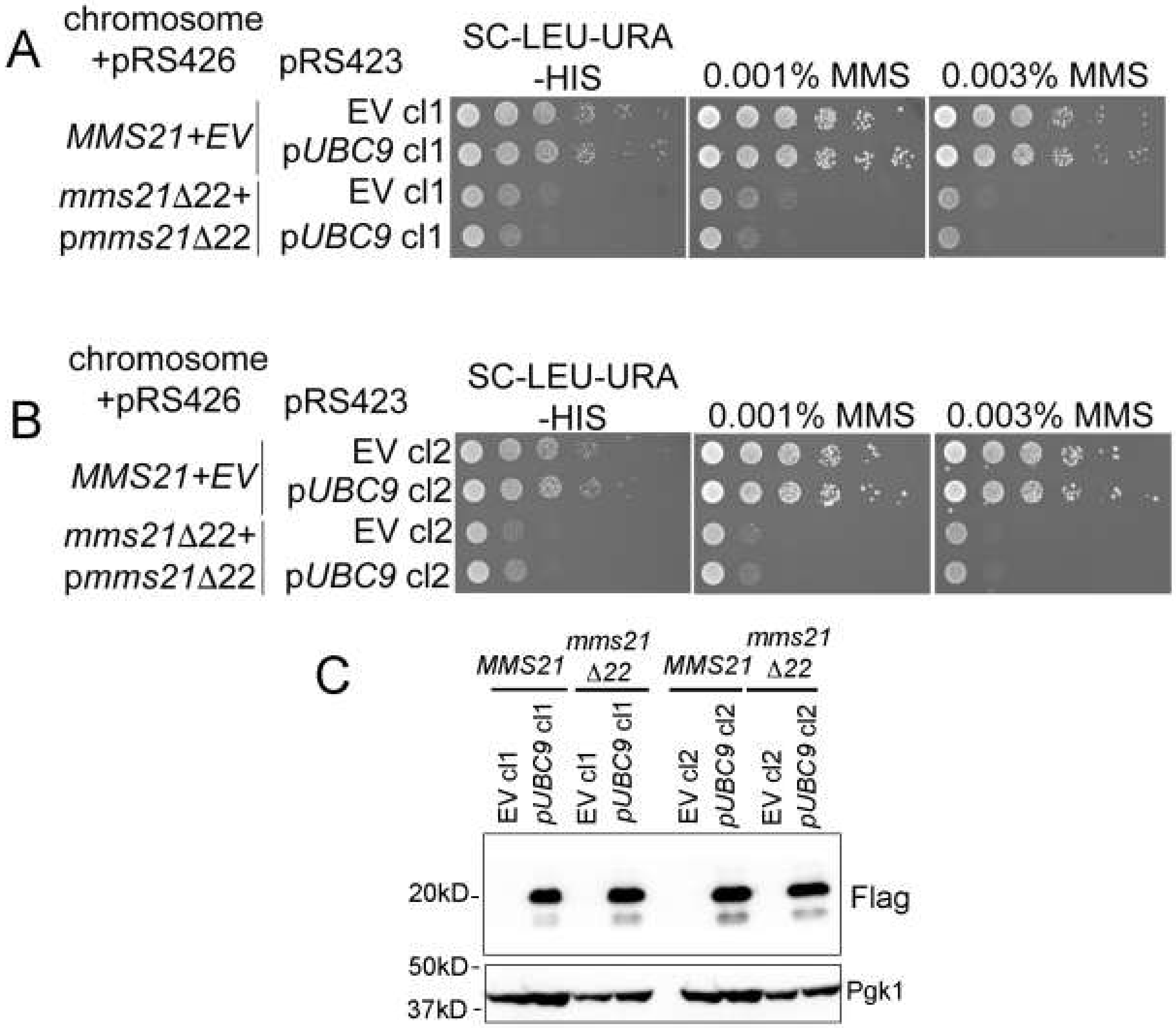

Supplemental Figure 3. Ubc9 overexpression in *mms21Δ22* mutants does not rescue the phenotypes of the mutants. **A-B)** Ubc9 was overexpressed from pRS423 in wild-type with empty vector (EV) or *mms21Δ22* mutants with *mms21Δ22* overexpression from pRS426. Successive 10-fold serial dilutions of indicated strains grown on SC-LEU-URA-HIS without or with different concentrations of MMS (n = 1 for each clone). **C)** Western blot analysis of Ubc9 epitope-tagged with Flag at the C-terminus in indicated strains. Pgk1 was a loading control. (cl. = clones)

Supplementary Materials

Manuscript title: Ligase-dependent and independent functions of the C-terminus of Mms21 contribute to optimal growth and genome stability in *Saccharomyces cerevisiae*

Description of Supplemental Figures 1C-F

To integrate the proposed mutants at the endogenous locus, we linearized the integration vector pRS405 with the 3' end of the *MMS21* gene carrying the mutation or the wild-type sequence and transformed it into cells (Romanos *et al.*, 1992). Clones were selected in SC-LEU media, followed by confirmation with PCR and sequencing (details are described in the Materials and Methods). Using this method resulted in tandem duplication of the sequence we introduced (Suppl. Figure 1C) (Romanos *et al.*, 1992). Although the partial sequence outside of the endogenous locus did not express, the availability of this copy made it possible for the vector to be lost via recombination (Suppl. Figure 1C) (Romanos *et al.*, 1992). However, the loss of the integration vector is generally rare – less than 1% per generation in the absence of selection has been reported (Romanos *et al.*, 1992). To prevent the loss of the transgene as part of the integration vector, we performed all experiments (both untreated and MMS treated conditions) in SC-LEU media (i.e., under the selection pressure).

Using the validated strains, we observed that *mms21Δ22* mutant clones exhibited growth defect under unchallenged conditions and were highly sensitive to the alkylating agent, MMS (Suppl. Figure 1D). However, the extent to which they showed negative phenotypes varied. Using PCR primer pairs that exclusively detected the wild-type or the transgene, we found that cl.10, a clone with better growth, but not cl.3, contained revertants (Suppl. Figure 1D). This observation suggests that a sub-population of cl.10 lost the integration vector along with the selectable marker, the *LEU*2 gene, and restored the genetic sequence encoding for the C-terminus even in the presence of the selection pressure (i.e., growth on SC-LEU plates) (Suppl. Figures 1C and D).

In theory, when the selection pressure is applied, loss of the integration vector should be suppressed (Romanos *et al.*, 1992). However, it has been well-documented that auxotrophs can utilize excess metabolites produced by prototrophs when both types of cells were grown together under selection pressure in a phenomenon called, "cross-feeding" (Hu *et al.*, 2024). We reasoned that "cross-feeding" and slow growth of prototrophic *mms21Δ22* mutants with the *LEU2* gene explained the appearance of auxotrophic wild-type cells within the population (Suppl. Figure 1D). We did not observe the loss of the integration plasmid in wild-type controls or *mms21-CH* mutants, further confirming that the *mms21Δ22* mutation was especially unfavorable for growth (Suppl. Figure 1D).

The growth disadvantage associated with the *mms21Δ22* mutation was further confirmed by cl.3 – although a population of this clone initially did not show revertants, we detected the presence of revertants in a later generation of cl.3 (compare Suppl. Figure 1D, performed at an earlier time point to Suppl. Figure 1E, performed at a later time point). The reversion phenotype coincided with improved growth and MMS-sensitivity of cl.3 (Suppl. Figure 1E). It is noteworthy that the *mms21Δ22* mutant population without revertants exhibited growth phenotype and MMS-sensitivity that were worse than ligase-deficient *mms21-CH* mutants (Suppl. Figure 1D). Unlike *mms21Δ22* mutants, *mms21Δ18* or *mms21sl* mutants did not show any observable defects and retained the transgene (Suppl. Figure 1F).

Table 1. Yeast strain list

Strain Name	Genotype	Source
BY4741	*MATa his3Δ1 leu2Δ0 met15Δ0 ura3Δ0*	Horizon Discovery
**Strains generated using the integration plasmid pRS405**		
y110	*BY4741 MMS21::mms21sl LEU2* cl. 1	This study
y111	*BY4741 MMS21::mms21sl LEU2* cl. 2	This study
y160	BY4741 *MMS21::MMS21 LEU2* cl.1	This study
y161	BY4741 *MMS21::MMS21 LEU2* cl.2	This study
y162	BY4741 *MMS21::mms21-CH LEU2* cl.1	This study
y163	BY4741 *MMS21::mms21-CH LEU2* cl.2	This study
y173	BY4741 *MMS21::mms21Δ18 LEU2* cl.1	This study
y174	BY4741 *MMS21::mms21Δ18 LEU2* cl.2	This study
y334	BY4741 *MMS21::mms21Δ22 LEU2* cl.3	This study
y335	BY4741 *MMS21::mms21Δ22 LEU2* cl.10	This study
**Strains generated by 2-step PCR homologous recombination**		
y368	*BAR1::NatMX MMS21::MMS21_3HA LEU2* cl.1	This study
y369	*BAR1::NatMX MMS21::MMS21_3HA LEU2* cl.2	This study
y371	*BAR1::NatMX MMS21::mms21Δ18_3HA LEU2* cl.1	This study
y372	*BAR1::NatMX MMS21::mms21Δ18_3HA LEU2* cl.2	This study
y360	*BAR1::NatMX MMS21::mms21Δ22_3HA LEU2* cl.3	This study
y361	*BAR1::NatMX MMS21::mms21Δ22_3HA LEU2* cl.4	This study
y362	*BAR1::NatMX MMS21::mms21Δ22_3HA LEU2* cl.5	This study
y363	*BAR1::NatMX MMS21::mms21Δ22_3HA LEU2* cl.1	This study
y364	*BAR1::NatMX MMS21::mms21Δ22_3HA LEU2* cl.2	This study
y365	*BAR1::NatMX MMS21::mms21Δ22_3HA LEU2* cl.6	This study
y366	*BAR1::NatMX MMS21::mms21Δ22_3HA LEU2* cl.7	This study
y367	*BAR1::NatMX MMS21::mms21Δ22_3HA LEU2* cl.8	This study
y385	*BAR1::NatMX MMS21::mms21_4A_3HA LEU2* cl.1	This study
y386	*BAR1::NatMX MMS21::mms21_4A_3HA LEU2* cl.2	This study
y377	*BAR1::NatMX MMS21::mms21sl_3HA LEU2* cl.1	This study
y378	*BAR1::NatMX MMS21::mms21sl_3HA LEU2* cl.2	This study
y390	*BAR1::NatMX MMS21::MMS21_3HA LEU2* pRS426 cl.1	This study
y391	*BAR1::NatMX MMS21::MMS21_3HA LEU2* pRS426 cl.2	This study
y398	*BAR1::NatMX MMS21::mms21Δ18_3HA LEU2* pRS426 cl.1	This study
y399	*BAR1::NatMX MMS21::mms21Δ18_3HA LEU2* pRS426 cl.2	This study
y392	*BAR1::NatMX MMS21::mms21Δ22_3HA LEU2* pRS426 cl.1	This study
y393	*BAR1::NatMX MMS21::mms21Δ22_3HA LEU2* pRS426 cl.2	This study
y394	*BAR1::NatMX MMS21::mms21Δ22_3HA LEU2* pRS426 *MMS21_3HA* cl.1	This study
y395	*BAR1::NatMX MMS21::mms21Δ22_3HA LEU2* pRS426 *MMS21_3HA* cl.2	This study
y396	*BAR1::NatMX MMS21::mms21Δ22_3HA LEU2* pRS426 *mms21Δ22_3HA* cl.1	This study
y397	*BAR1::NatMX MMS21::mms21Δ22_3HA LEU2* pRS426 *mms21Δ22_3HA* cl.2	This study

y404	*BAR1::NatMX MMS21::mms21Δ22_3HA LEU2* pRS426 *mms21Δ22_3HA* cl.3	This study
y400	*BAR1::NatMX MMS21::mms21-CH_3HA LEU2* cl.1	This study
y401	*BAR1::NatMX MMS21::mms21-CH_3HA LEU2* cl.2	This study
y381	*BAR1::NatMX MMS21::mms21Nterm_3HA LEU2* cl.1	This study
y382	*BAR1::NatMX MMS21::mms21Nterm_3HA LEU2* cl.2	This study
y427	*BAR1::NatMX MMS21::mms21-CHΔ22_3HA LEU2* cl.1	This study
y428	*BAR1::NatMX MMS21::mms21-CHΔ22_3HA LEU2* cl.2	This study
y443	*BAR1::NatMX MMS21::MMS21_3HA LEU2* pRS426 *MMS21_3HA* cl1	This study
y444	*BAR1::NatMX MMS21::MMS21_3HA LEU2* pRS426 *MMS21_3HA* cl2	This study
y445	*BAR1::NatMX MMS21::MMS21_3HA LEU2* pRS426 *mms21Δ22* cl1	This study
y446	*BAR1::NatMX MMS21::MMS21_3HA LEU2* pRS426 *mms21Δ22* cl2	This study
y412	*BAR1::NatMX MMS21::mms21-CH_3HA LEU2* pRS426 cl1	This study
y447	*BAR1::NatMX MMS21::mms21-CH_3HA LEU2* pRS426 *MMS21_3HA* cl1	This study
y449	*BAR1::NatMX MMS21::mms21-CH_3HA LEU2* pRS426 *mms21Δ22_3HA* cl1	This study
y450	*BAR1::NatMX MMS21::mms21-CH_3HA LEU2* pRS426 *mms21Δ22_3HA* cl2	This study
y452	*BAR1::NatMX MMS21::MMS21_3HA LEU2* pRS426 *mms21-CH_3HA* cl1	This study
y453	*BAR1::NatMX MMS21::MMS21_3HA LEU2* pRS426 *mms21-CH_3HA* cl2	This study
y455	*BAR1::NatMX MMS21::mms21Δ22_3HA LEU2* pRS426 *mms21-CH_3HA* cl1	This study
y456	*BAR1::NatMX MMS21::mms21Δ22_3HA LEU2* pRS426 *mms21-CH_3HA* cl2	This study
y439	*BAR1::NatMX MMS21::MMS21_3HA LEU2* pRS426 pRS423 cl.1	This study
y440	*BAR1::NatMX MMS21::MMS21_3HA LEU2* pRS426 pRS423 cl.2	This study
y435	*BAR1::NatMX MMS21::MMS21_3HA LEU2* pRS426 pRS423 *UBC9_Flag* cl.1	This study
y436	*BAR1::NatMX MMS21::MMS21_3HA LEU2* pRS426 pRS423 *UBC9_Flag* cl.2	This study
y441	*BAR1::NatMX MMS21::mms21Δ22_3HA LEU2* pRS426 *mms21Δ22_3HA* pRS423 cl.1	This study
y442	*BAR1::NatMX MMS21::mms21Δ22_3HA LEU2* pRS426 *mms21Δ22_3HA* pRS423 cl.2	This study
y437	*BAR1::NatMX MMS21::mms21Δ22_3HA LEU2* pRS426 *mms21Δ22_3HA* pRS423 *UBC9_Flag* cl.1	This study
y438	*BAR1::NatMX MMS21::mms21Δ22_3HA LEU2* pRS426 *mms21Δ22_3HA* pRS423 *UBC9_Flag* cl.2	This study

Table 2: Primer list

**Name**	**Sequence**
mms21_F	cgaggtcgacggtatcgataagctttacctgggataaatatcgtac
mms21_R	cggccgctctagaactagtggatcctcataaaacatcgatggc
mms21-d22_R	cggccgctctagaactagtggatcctcaacaccttagttccataatag
mms21_d18_R	cggccgctctagaactagtggatcctcacttggcgatcttacac
mms21-simless_R	cggccgctctagaactagtggatcctcaggcttgactacttcttttatc
5pRS316	gatgtgctgcaaggcgattaagttg
3pRS400	cacaggaaacagctatgaccatg
5HindIII_mms21_321	atcgataagctttacctgggataa
3MMS21-no stop-NheI-1HA	ata gtc agg aac atc gta tgg gta aaa cgc tag cgg taaaacatcgatggcttgact
3mms21d18-no stop-NheI-1HA	ata gtc agg aac atc gta tgg gta aaa cgc tag cgg cttggcgatcttacaccttag
3mms21d22-no stop-NheI-1HA	ata gtc agg aac atc gta tgg gta aaa cgc tag cgg acaccttagttccataatagg
3mms21sl-no stop-NheI-1HA	ata gtc agg aac atc gta tgg gta aaa cgc tag cgg ggcttgactacttcttttatc
3mms21Nterm-no stop-NheI-1HA	ata gtc agg aac atc gta tgg gta aaa cgc tag cgg caattcaattttaccaccttc
3_2HA	cga tcc tgc ata gtc cgg gac gtc ata ggg ata gcc cgc ata gtc agg aac atc gta tgg
3_3HA-STOP-BamHI	cgc gga tcc tta agc gta atc tgg aac gtc ata tgg ata cga tcc tgc ata gtc cgg gac
5Mms21_400	ctgcaaccat ggttaataac
3pRS40x_3HA	gtatggtgcactctcagtacaatct ttaagcgtaatctggaacgtcata
5pRS40x_2step	agattgtactgagagtgcaccatac
40dnMms21_LEU2_r	gggccgaagggctcggataagagaaacaataattttgttt ctg tgc ggt att tca cac cg
3LEU2_880pRS405	atgccacggttctgctccag
Mms21_50up	cagttaaagaaaccaaggca
Mms21_56dn	gcccgaagtt caagcaaagc
HindIII_yMMS21_f	cgaggtcgacggtatcgataagcttatggccttgaacgataatc
MMS21_NotI_r	tggagctccaccgcggtggcggccgcttaagcgtaatctggaac
5Ubc9_ex2	aa aaaaatggag aaaggatcat
3FlagUbc9_noSTOP	tcatgatctttataatcaccgtcatggtctttgtagtctttagagtactgtttagcttga
5Ubc9_ex1	atgagtagtttgtgtctacagcgtcttcaggaagaaagaa aaaaatggag aaaggatcat
3STOP_Flag	ctacttgtcatcgtcatccttgtaatcgatatcatgatctttataatcacc

5pRS423_EcoRI_Ubc9	tatcgataagcttgatatcgaattcatgagtagtttgtgtctac
Bar1KONat_f	atcataccaa aataaaaaga gtgtctagaa gggtcatata cggatccccgggttaattaa
Bar1KONat_r	tgatatttatatgctataaagaaattgtactccagatttc gaattcgagctcgtttaaac
Bar1_120up	gaaagcacgt cgagccttgt
Bar1_166dn	gctacttgttcaaaattgtg

References

1. Hu, K. K. Y., Suri, A., Dumsday, G., & Haritos, V. S. (2024). Cross-feeding promotes heterogeneity within yeast cell populations. *Nat Commun*, *15*(1), 418. https://doi.org/10.1038/s41467-023-44623-y
2. Romanos, M. A., Scorer, C. A., & Clare, J. J. (1992). Foreign gene expression in yeast: a review. *Yeast*, *8*(6), 423-488. https://doi.org/10.1002/yea.320080602
