# Supplementary material for: Ligase-dependent and independent functions of the C-terminus of Mms21 contribute to optimal growth and genome stability in Saccharomyces cerevisiae
Source: Mol Biol Cell. 2026 Apr 8;37(6):ar50. doi: 10.1091/mbc.E25-11-0567 (PMC13200702; doi:10.1091/mbc.E25-11-0567)
Supplement: Supplementary file 2 [file reviewer_reportE25-11-0567.pdf]

Ligase-dependent and independent functions of the C-terminus of Mms21 contribute to optimal growth and genome stability in Saccharomyces cerevisiae

Yee Mon Thu, Cheung Li, Anny Vo, and Nkechinye Baadi

Corresponding author(s): Yee Mon Thu, Colby College

Review Timeline:

Submission Date:	2025-11-29
Editorial Decision:	2026-01-13
Revision Received:	2026-03-15
Accepted:	2026-03-31

Editor-in-Chief: John Aitchison

Transaction Report:

(Note: With the exception of the correction of typographical or spelling errors that could be a source of ambiguity, letters and reports are not edited. The original formatting of letters and referee reports may not be reflected in this compilation.)

RE: Manuscript #E25-11-0567
TITLE: Ligase-dependent and independent functions of the C-terminus of Mms21 contribute to optimal growth and genome stability in Saccharomyces cerevisiae

Dear Dr. Thu:

Thank you for submitting your paper entitled "Ligase-dependent and independent functions of the C-terminus of Mms21 contribute to optimal growth and genome stability in Saccharomyces cerevisiae" to MBoC. Two experts in the field have now evaluated your manuscript. Both Reviewers agree that for the manuscript to be suitable for publication will require various revisions and their reexamination. Among the concerns of both Reviewers are the overall breadth and clarity of the manuscript. They provide specific recommendations for improving the manuscript in these general areas, which I also feel are important. For example, to improve the comprehensiveness of the study, Reviewer 1's recommendations include examining the subcellular localization of key mms21 mutants and their impact on protein sumoylation. Reviewer 2 makes several excellent suggestions including to consider using the alpha fold structure of Mss21 to assess structure-function relationships of selected mutants. Both Reviewers also stress similar concerns with content of the Results section and the overall clarity of the manuscript. Please address these and other points raised by the Reviewers in a revised version of your manuscript. I also ask that you provide a point-by-point response to the Reviewers' comments, and indicate how you have incorporated your responses into the revised manuscript. I look forward to seeing your updated manuscript.

Sincerely,

Richard Wozniak
Monitoring Editor
Molecular Biology of the Cell

Dear Dr. Thu,

The review of your manuscript, referenced above, is now complete. The Monitoring Editor has decided that your manuscript is not acceptable for publication at this time, but may be deemed acceptable after specific revisions are made, as described in the Monitoring Editor's decision letter above and the reviewer comments below.

A reminder: Please do not contact the Monitoring Editor directly regarding your manuscript. If you have any questions regarding the review process or the decision, please contact the MBoC Editorial Office (mboc@ascb.org).

When submitting your revision include a rebuttal letter that details, point-by-point, how the Monitoring Editor's and reviewers' comments have been addressed. (The file type for this letter must be "rebuttal letter"; do not include your response to the Monitoring Editor and reviewers in a "cover letter.") Please bear in mind that your rebuttal letter will be published with your paper if it is accepted, unless you have opted out of publishing the review history.

Authors are allowed 90 days to submit a revision. If this time period is inadequate, please contact us at mboc@ascb.org.

Revised manuscripts are assigned to the original Monitoring Editor whenever possible. However, special circumstances may preclude this. Also, revised manuscripts are often sent out for re-review, usually to the original reviewers when possible. The Monitoring Editor may solicit additional reviews if it is deemed necessary to render a completely informed decision.

In preparing your revised manuscript, please follow the instruction in the Information for Authors (www.molbiolcell.org/info-for-authors). In particular, to prepare for the possible acceptance of your revised manuscript, submit final, publication-quality figures with your revision as described.

To submit the rebuttal letter, revised manuscript, and figures, use this link: Link Not Available

MBoC offers the option to publish your work with immediate open access. Open access can increase the discoverability and usability of your research. If you would like to publish your paper with immediate open access but did not select this option during initial submission, please contact the editorial office at mbc@ascb.org.

Authors of Articles and Brief Communications are also encouraged to create a short video abstract to accompany their article when it is published. These video abstracts, known as Science Sketches, are up to 2 minutes long and will be published on YouTube and then embedded in the article abstract. Science Sketch Editors on the MBoC Editorial Board will guide you as you

prepare your video. Information about how to prepare and submit a video abstract is available at www.molbiolcell.org/science-sketches. Please contact mboc@ascb.org if you are interested in creating a Science Sketch.

Sign up for MBoC's new content alerts here https://tinyurl.com/3bcsyk3e

Thank you for submitting your manuscript to MBoC. We look forward to receiving your revised paper.

Sincerely,

Eric Baker
Journal Production Manager
MBoC Editorial Office
mbc@ascb.org

Reviewer #1 (Remarks to the Author):

The authors are analyzing C-terminal truncation mutants of the SUMO E3 ligase Mms21. Several mutants were tested: yeast mms21Δ22 (a hypomorphic alleles with intact E3 ligase domain that showed reduced protein levels), a comparator Δ18 (no phenotype), the ligase dead Mms21 mutant mms21-CH, and a few others. The yeast mms21Δ22 mutant corresponds to a human mms21 mutant p.Ala234Glufs*4 mutation, underscoring the relevance of this work. Using this approach, the authors sought to compare and contrast ligase-dependent and independent effects of the mutants and specifically their effect on genotoxic insults (0.01-0.02% MMS, HU, UV, etc). Notably, as previously determined, defects in Mms21 E3 ligase activity are not essential but result in sensitivity to genotoxic agents. The same seems to be true for the Δ22 mutation, but not the Δ18 mutant, which leaves a conserved "I" residue intact. The ligase dead mms21-CH mutant shows less severe phenotypes when compared to Δ22, both untreated or when subjected to genotoxic stressors. Combining mms21-CH/Δ22 in yeast cells reveals a slow growth phenotype identical to Δ22. However, the finding that complete deletion of the RING domain and C-terminus (mms21Nterm (A183-267)) fares better than a Δ22 mutant suggests a dominant-negative scenario. However, there is no clear dominant-negative interaction observed when Δ22 is expressed in the mms21-CH mutant strain. The localization and sumoylation activity of the Δ22 mutants were not tested. So, while ultimately this study falls short on explaining the phenotype of the Δ22 mutants, this study is an important resource and step towards a better understanding of Mms21's structure and function.

Major:

• It might be helpful to revise the abstract to better highlight which mutants were tested and the key findings, as this will make the main points clearer. (also see the minor points below). I struggled to get a clear understanding of the study by simply reading the abstract, but it became clear past Figure 1.

Section starting with "To overcome the challenge of reversion ..."and related Fig 2 (pg): I suggest deleting Fig 1D-G. While I appreciate that much work has gone into the analysis of revertants, I think this is confusing for a non-yeast audience, adds unknowns re. potential expression of Mms2 fragments, and dilutes the message conveyed by the stable integrants in Fig. 2. The authors could consider cutting the paragraph associated with Fig. 1D-G and moving it to supplementary data. If the authors have additional data on the frequency of reversion, that would be of interest.

Though not entirely clear, it seems that the HA-tag on mms21Δ22 seems to be added at the C terminus of the truncation. (M&M: ... mms21d22-no stop-NheI-1HA). The authors should clarify where the epitope tag resides and whether there could be an unintended effect due to the epitope tag at the C-terminal domain that is being scrutinized. A previous study showed that Mms21 with a C-terminal myc tag has a slight growth defect: https://pmc.ncbi.nlm.nih.gov/articles/PMC5570088/.

• Two important aspect of the observed Mms21 mutations that were not inspected in this study are their subcellular localization and involvement in sumoylation of proteins (e.g. Smc5) (PMCID: PMC555716). For example, the surprisingly low levels of mms21Δ22 may be due to its mislocalization. Similarly, it would be interesting to determine if Δ22 results in a lack of sumoylation, which is linked to aberrant nucleolar structure in a mms21-11 mutant. These analyses may be beyond the scope of this study, but the authors should discuss the caveat that mutant protein localization and sumoylation activity are affected in the Δ22 mutants. Also Fig 4G should be removed as it does not add to our understanding.

Minor:

• Abstract: ... We modeled a mutant of MMS21, associated with a rare genetic
human condition characterized by high genome instability, in Saccharomyces cerevisiae. - Initially, it is not clear whether this is a

yeast or a human Mms21 that's been tested here. Can the authors state: We modeled a mutant of human/yeast MMS21 ... or Hs/ScMms21? The significant statement could help clarify this.

• Abstract: This mutation C-terminally truncated the Mms21 protein, without affecting the residues in the E3 ligase domain. Thus, we hypothesized that the C-terminus regulated ligase-independent functions of Mms21. Similar to the ligase-deficient mutant, the C-terminal truncation mutant of yeast Mms21, mms21 22, exhibited a G2/M delay during normal growth.
-> this is confusing bc the authors first state that the residues in the ligase domain are not affected, BUT then continue to state" .... similar to the ligase-deficient mutant !? The authors should clearly state which mutants are tested and whether a mutant with intact Mms21 E3 is ligase-deficient or not. This becomes clear in the reminder of the manuscript but should be rephrased to improve clarity in the abstract.

• Introduction. - It is not clear what " prone to ... chemical alterations" refers to? Do you mean sensitive to genotoxic stressors?

• The significance of Mms21's activity in genome integrity is further underscored by the identification of mis-regulated Mms21's activities in human patients - do the authors provide details of associated phenotypes and disease? They state: ... a human rare genetic condition, a NSMCE2 (the human gene of MMS21) variant designated as p.Ala234Glufs*4 was identified (Payne et al., 2014). But the human disease (developmental delay?) is not described beyond the molecular level.

• Introduction: The Smc5/6/Mms21 complex enhances the dissolution activity of the STR complex by promoting Sgs1 sumoylation, a process important for the assembly of the STR. - do you mean "important for STR complex dynamics".

• Fig 3C: The authors study the progression of their untreated mutants from "Asyn into S phase". I am wondering why they did not first arrest their cells using alpha factor and then release them into the cell cycle. This seems like it may be a better way to study a potential cell cycle progression phenotype? Considering that the Δ22 mutants are HU sensitive, it could also be informative to see how the cell cycle profiles react in response to HU-induced arrest.

• As stated, Mms21 is a subunit of the chromatin-associated Smc5/6 (structural maintenance of chromosome 5/6) complex that regulates DNA replication, repair, recombination, and chromosome segregation - do the authors test all of the above? I understand that the present analysis goes beyond the scope of this paper, but I think that a caveat needs to be added in the discussion that not all possible phenotypic assays associated with the Δ22 and human mutant were explored.

Reviewer #2 (Remarks to the Author):

Li et al. present a study examining the role of the c-terminus of the SUMO E3 ligase, Mms21. This protein has a conserved role in the maintenance of genome integrity as a component of the Smc5/6 complex, which is involved in resolving DNA intermediates following recombination. There is a rare genetic condition in humans caused by a mutation in the human homolog of Mms21, NSMCE2, that truncates the C-terminus of the protein. The authors created analogs to the human genes/proteins, mms21 Δ18, which ends at the same point as the human gene and lacks the C-terminal SIM present in the yeast gene, and mms21 Δ22, which ends at a point equivalent to the human disease mutant. They find that the human-like truncation (Δ18) is well tolerated in yeast, leading to no growth defects in the presence of DNA-damaging agents relative to WT Mms21. However, the disease mutant analog (Δ22) leads to compromised growth under the same DNA-damaging conditions. They find that yeast expressing the disease mutant have lower levels of mms21 protein, but that this does not appear to be the reason for their growth defects. Instead, it appears that the C-terminus both promotes ligase activity, and is also necessary for non-ligase activities of the protein.

The use of yeast Mms21 to investigate a human disease mutation is clever, and the work is carefully done, however there are problems that stop it from being suitable for publication in MBoC in its current form, primarily because the complex interplay of mutants and phenotypes used to derive the conclusions are difficult to follow, and so it needs significant rewriting to develop the clarity necessary. Some of this clarity in story telling may be facilitated by considering the predicted protein structure, which I think will improve the ways that the authors think and talk about their work. I believe the authors can likely revise the manuscript to address these concerns.

Major Issues
1. The authors should examine the alpha fold structure prediction for Mms21 (which is high confidence and has little IDR). The entire study makes sense in light of the predicted structure. The mms21 Δ18 truncation removes a c-terminal tail that sticks out from the structure. The residues that are present in Δ18, but removed in Δ22 contact both the N-terminus and the SP-RING domain, and appear to hold the whole protein together. Thus the disease mutant likely disrupts both SP-RING positioning or stability, as well as structural roles that Mms21 plays in the larger Smc5/6 complex. Incorporating an annotated structure to examine how the deleted regions are positioned relative to the N-terminus and the SP-RING would go a long way towards understanding what is happening here. Whether this is better at the beginning or the end, I leave up to the authors. I could see advantages of either organization. Without this readily available information, the discussion feels unanchored. I think considering this structure would change that.
2. The story is hard to follow. I believe part of this is the complexity of the mms21 mutant naming conventions, which are hard to

keep track of. Descriptive text (such as I used above) when talking about mms21Δ18 vs mms21Δ22 would help the reader greatly. Another issue is that the authors set up a nice problem of the disease mutant in the introduction, but don't center their conclusions on that. They find that the truncation similar to WT human NSMCE2 does not disrupt function in yeast (at least appreciably), but that the disease mutant analog does. This framing is essentially abandoned after the introduction, but is a very useful way to think about it. Successfully modeling a human disease mutant in yeast is great, the authors should make it more clear that they have succeeded in doing this.
3. The explanation of the truncation effect on ligase-dependent function vs ligase-independent function was convoluted and required a lot of work on my part to understand. I ultimately came to the conclusion that the authors were warranted in their conclusions, but it was not easy. Given the centrality of this idea to their overall conclusions, this should be made much more clear. A cartoon summarizing the logic in this section might help.
4. With regards to the mutant nomenclature, a cartoon like the one in 4B would be helpful earlier in the paper, and containing all mutants used.
5. I don't find the missense analysis adds anything to the study. In fact, I think it serves as a distraction from the more relevant idea that you have a disease mutant that is informing your mutagenesis strategy. The missense analysis leads the reader to think there will examination of point mutants in I235, and that never happens. Instead it is ignored and they move back to the truncations that are justified by the human protein.
6. The authors point out that the SP-RING is required for ligase activity, but is the SP-RING sufficient for ligase activity? Is the C-terminus a required part for ligase function in vitro, or is its requirement solely in the context of the larger Smc complex?
7. Is the Δ22 dominant negative? The experiments with adding Δ22 on a plasmid do not test the impact of adding it to a WT background. The authors mention as a shortcoming that previous work only examined the human c-terminal truncation in the context of the WT protein. Testing this directly in yeast would add a lot to our understanding of the impact of this mutation.
8. The problem solving with the integrating plasmid in the beginning is well done, but is a distraction from the scientific questions being asked. The authors may want to consider moving that section to the supplement, and covering what they did in broad strokes in a sentence or two and then move on to the experiments that effectively test Mms21 function.
9. There is no reference to the number of replicates of the spotting assays.
10. The western blotting used to assess mms21Δ22 levels should be quantified, analyzed, and the number of replicates listed in the figure legends.
Minor Issues
1. The first "significance statement" doesn't read quite right, I think it's the middle sentence coupled with "to this end" at the beginning of the next. The other bullet points are fine, so I think the authors just need to iterate on this one.
2. The time needed for arrest in alpha factor seems long. I assume these experiments were done in bar1Δ cells (especially given the low alpha factor concentration used)? There is mention of deleting BAR1 in the methods.
3. In the second-to-last paragraph in the results, the authors start with "In agreement with the data in Figures 1E and F..." This requires the reader to either remember that data, or stop and go back to look at the figure, when the authors could just say what their new experiments are agreeing with.

Colby

COLBY COLLEGE
4000 Mayflower Hill
Waterville, Maine 04901
TEL 207-859-4000

March 15, 2026

Dear Dr. Wozniak,

Thank you for the summary of the suggestions. We also thank the reviewers for their constructive feedback, which helped improve the clarity, biological significance, and logical flow of the manuscript.

We have revised the manuscript according to the suggestions. Figures 1, 2, and 4 have been modified and reorganized. Figure 5 is a new model figure. Supplemental Figure 1 has been modified.

As suggested by the MBoC editorial office, the revised manuscript includes yellow highlights that indicate major changes according to the reviewers' suggestions.

Point-by-point responses are included below.

Thank you for your time and help.

Sincerely,

(Yee Mon Thu)

Yee Mon Thu, PhD

Assistant Professor
Department of Biology
Colby College
5700 Mayflower Hill Dr
Waterville ME 04901
Ph: 207-859-5731

Response to the Reviewers

RE: Manuscript #E25-11-0567
TITLE: Ligase-dependent and independent functions of the C-terminus of Mms21 contribute to optimal growth and genome stability in Saccharomyces cerevisiae

Dear Dr. Thu:

Thank you for submitting your paper entitled "Ligase-dependent and independent functions of the C-terminus of Mms21 contribute to optimal growth and genome stability in Saccharomyces cerevisiae" to MBoC. Two experts in the field have now evaluated your manuscript. Both Reviewers agree that for the manuscript to be suitable for publication will require various revisions

Colby COLBY COLLEGE
4000 Mayflower Hill
Waterville, Maine 04901
TEL 207-859-4000

and their reexamination. Among the concerns of both Reviewers are the overall breadth and clarity of the manuscript. They provide specific recommendations for improving the manuscript in these general areas, which I also feel are important. For example, to improve the comprehensiveness of the study, Reviewer 1's recommendations include examining the subcellular localization of key mms21 mutants and their impact on protein sumoylation. Reviewer 2 makes several excellent suggestions including to consider using the alpha fold structure of Mss21 to assess structure-function relationships of selected mutants. Both Reviewers also stress similar concerns with content of the Results section and the overall clarity of the manuscript. Please address these and other points raised by the Reviewers in a revised version of your manuscript. I also ask that you provide a point-by-point response to the Reviewers' comments, and indicate how you have incorporated your responses into the revised manuscript. I look forward to seeing your updated manuscript.

Sincerely,

Richard Wozniak
Monitoring Editor
Molecular Biology of the Cell

Thank you for thoughtful suggestions. We greatly appreciate the editor’s and reviewers’ thoughts on how to make this a better-quality manuscript. Original comments are in black, and our responses are in blue.

Reviewer #1 (Remarks to the Author):

The authors are analyzing C-terminal truncation mutants of the SUMO E3 ligase Mms21. Several mutants were tested: yeast mms21∆22 (a hypomorphic alleles with intact E3 ligase domain that showed reduced protein levels), a comparator ∆18 (no phenotype), the ligase dead Mms21 mutant mms21-CH, and a few others. The yeast mms21∆22 mutant corresponds to a human mms21 mutant p.Ala234Glufs*4 mutation, underscoring the relevance of this work. Using this approach, the authors sought to compare and contrast ligase-dependent and independent effects of the mutants and specifically their effect on genotoxic insults (0.01-0.02% MMS, HU, UV, etc). Notably, as previously determined, defects in Mms21 E3 ligase activity are not essential but result in sensitivity to genotoxic agents. The same seems to be true for the ∆22 mutation, but not the ∆18 mutant, which leaves a conserved "I" residue intact. The ligase dead mms21-CH mutant shows less severe phenotypes when compared to ∆22, both untreated or when subjected to genotoxic stressors. Combining mms21-CH/∆22 in yeast cells reveals a slow growth phenotype identical to ∆22. However, the finding that complete deletion of the RING domain and C-terminus (mms21Nterm (A183-267)) fares better than a ∆22 mutant suggests a dominant-negative scenario. However, there is no clear dominant-negative interaction observed when ∆22 is expressed in the mms21-CH mutant strain. The localization and sumoylation activity of the ∆22 mutants were not tested. So, while ultimately this study falls short on explaining the phenotype of the ∆22 mutants, this study is an important resource and step towards a better understanding of Mms21's structure and function.

Colby

COLBY COLLEGE
4000 Mayflower Hill
Waterville, Maine 04901
TEL 207-859-4000

Major:

• It might be helpful to revise the abstract to better highlight which mutants were tested and the key findings, as this will make the main points clearer. (also see the minor points below). I struggled to get a clear understanding of the study by simply reading the abstract, but it became clear past Figure 1.

We acknowledge that the original abstract should have contained more specifics about other mutants that we compared *mms21Δ22* with. The abstract has been revised according to the reviewer's suggestions, highlighting mutants tested and key findings. Due to the 200-word limit of the abstract, we had to prioritize mentioning of some mutants over the others.

Section starting with "To overcome the challenge of reversion ..."and related Fig 2 (pg): I suggest deleting Fig 1D-G. While I appreciate that much work has gone into the analysis of revertants, I think this is confusing for a non-yeast audience, adds unknowns re. potential expression of Mms2 fragments, and dilutes the message conveyed by the stable integrants in Fig. 2. The authors could consider cutting the paragraph associated with Fig. 1D-G and moving it to supplementary data. If the authors have additional data on the frequency of reversion, that would be of interest.

Thank you for the suggestion. Both reviewers pointed out that we should move the figures regarding revertants to the supplemental materials. In the revised manuscript, we summarized the data describing revertants to support the main messages but moved the details of the description to the supplemental materials. Original Fig. 1D-G are now Suppl. Fig.1C-F.

To maintain the logical progression of the story, we included Fig2B in the revise manuscript to make comparison between *mms21-CH* and *mms21Δ22* mutants.

Though not entirely clear, it seems that the HA-tag on mms21Δ22 seems to be added at the C terminus of the truncation. (M&M: ... mms21d22-no stop-NheI-1HA). The authors should clarify where the epitope tag resides and whether there could be an unintended effect due to the epitope tag at the C-terminal domain that is being scrutinized. A previous study showed that Mms21 with a C-terminal myc tag has a slight growth defect: https://pmc.ncbi.nlm.nih.gov/articles/PMC5570088/.

This is an important point that we also considered.

In the revised manuscript, we indicated that the 3HA tag is at the C-terminus of the protein in the results section (p.5).

"We integrated *MMS21, mms21Δ18*, *mms21Δ22*, or *mms21-CH* alleles at the endogenous locus along with the *LEU2* selectable marker. Wild-type and mutant proteins generated by this method contain a 3HA epitope tag at the C-terminal end of the protein."

We did not observe any noticeable difference between untagged and tagged strains. The strains used in Figure 1 of the original manuscript (Suppl. Fig. 1C-F in the revised manuscript) do not have 3HA tags. They show comparable growth to strains with the 3HA tag (e.g. strains in Figure 2 of the original manuscript and the revised manuscript). In the revised manuscript, we compared strains with and without 3HA on the same plates and included the data as Suppl. Figure 1G.

Colby

COLBY COLLEGE
4000 Mayflower Hill
Waterville, Maine 04901
TEL 207-859-4000

• Two important aspect of the observed Mms21 mutations that were not inspected in this study are their subcellular localization and involvement in sumoylation of proteins (e.g. Smc5) (PMCID: PMC555716). For example, the surprisingly low levels of mms21∆22 may be due to its mislocalization. Similarly, it would be interesting to determine if ∆22 results in a lack of sumoylation, which is linked to aberrant nucleolar structure in a mms21-11 mutant. These analyses may be beyond the scope of this study, but the authors should discuss the caveat that mutant protein localization and sumoylation activity are affected in the ∆22 mutants. Also Fig 4G should be removed as it does not add to our understanding.

We appreciate the reviewer's excellent suggestions and acknowledge that our data are limited to genetic evidence. As stated by the reviewer, determining the subcellular localization and sumoylation of Mms21's substrates are beyond the scope of our study. For example, we have generated Smc5-Flag-8His tagged strains in *MMS21*, *mms21-CH*, and *mms21∆22* backgrounds. We have started troubleshooting to detect sumoylated Smc5 in these genotypes in the presence of a DNA damaging agent, MMS. We currently faced two major technical challenges. First, we will need to collect enough cellular extract to be able to detect sumoylated Smc5 - sumoylated proteins are generally low in abundance (e.g. (Thu *et al.*, 2016)). Second, *mms21∆22* mutants are highly sensitive to MMS (e.g. Figure 2B in the revised manuscript). We are aiming for the optimal condition in which enough *mms21∆22* cells still grow to yield sufficient extract, but the concentration and time of treatment are efficacious enough to induce sumoylation of Smc5 in cells with proficient Mms21 E3 SUMO ligase activity. These experiments are still ongoing.

Thus, we included the limitations of our study in the discussion section to acknowledge that we did not explore some phenotypes associated with Mms21 defects (p. 9 and 12).

"Our conclusion about the contribution of the C-terminus to the ligase activity is mainly based on genetic evidence - we did not examine sumoylation of known substrates of Mms21, such as Smc5, in *mms21∆22* mutants. In human cells, the C-terminal truncation does not appear to significantly reduce the ligase activity of the protein (Payne *et al.*, 2014). It is noteworthy that the ligase activity of the human p.Ala234Glufs*4 Mms21 mutant was examined in a system in which both SUMO and the mutant Mms21 were overexpressed while the endogenous Mms21 was still present (Payne *et al.*, 2014). Further studies are necessary in both yeast and mammalian cells to better elucidate the role of the C-terminus in Mms21's ligase activity."

"We acknowledge that the scope of our study is limited to genetic evidence, suggesting potential mechanisms by which Mms21's C-terminus contributes to genome stability. Our study did not examine whether the lack of the C-terminus disrupts DNA replication or recombination, processes known to be regulated by Mms21 and the Smc5/6 complex (Peng *et al.*, 2023). G2/M delay phenotypes associated with the C-terminal truncation in *mms21∆22* mutants imply that these cells may experience replication stress and subsequent chromosome segregation errors. Likewise, we did not examine the localization of the C-terminally truncated Mms21 mutant. Direct biochemical or cellular assays are necessary to better understand the contributions of the C-terminus to Mms21's functions."

Figure 4G has been removed. We included Figure 5 to better illustrate the proposed ligase-dependent and -independent functions of Mms21.

Colby

COLBY COLLEGE
4000 Mayflower Hill
Waterville, Maine 04901
TEL 207-859-4000

Minor:

• Abstract: ... We modeled a mutant of MMS21, associated with a rare genetic human condition characterized by high genome instability, in Saccharomyces cerevisiae. - Initially, it is not clear whether this is a yeast or a human Mms21 that's been tested here. Can the authors state: We modeled a mutant of human/yeast MMS21 ... or Hs/ScMms21? The significant statement could help clarify this.

We revised the description to make it clear that we use the yeast gene in our study.

• Abstract: This mutation C-terminally truncated the Mms21 protein, without affecting the residues in the E3 ligase domain. Thus, we hypothesized that the C-terminus regulated ligase-independent functions of Mms21. Similar to the ligase-deficient mutant, the C-terminal truncation mutant of yeast Mms21, mms21 22, exhibited a G2/M delay during normal growth.
-> this is confusing bc the authors first state that the residues in the ligase domain are not affected, BUT then continue to state" .... similar to the ligase-deficient mutant !? The authors should clearly state which mutants are tested and whether a mutant with intact Mms21 E3 is ligase-deficient or not. This becomes clear in the reminder of the manuscript but should be rephrased to improve clarity in the abstract.

The abstract has been revised based on the reviewer's suggestion.

• Introduction. - It is not clear what " prone to ... chemical alterations" refers to? Do you mean sensitive to genotoxic stressors?

We realized that this was confusing. We changed the description in the revised manuscript.

• The significance of Mms21's activity in genome integrity is further underscored by the identification of mis-regulated Mms21's activities in human patients - do the authors provide details of associated phenotypes and disease? They state: ... a human rare genetic condition, a NSMCE2 (the human gene of MMS21) variant designated as p.Ala234Glufs*4 was identified (Payne et al., 2014). But the human disease (developmental delay?) is not described beyond the molecular level.

We agree that our main focus is at the molecular level. We included the statements describing Mms21's connection to human conditions to emphasize biological significance. Since our study could not address phenotypes at the organismal level, we did not describe the clinical phenotypes.

As per the reviewer's suggestion to make the significance more apparent, we now included a sentence in the introduction (p.4).

"Patients with compound these heterozygous mutations exhibited developmental delay and insulin resistance (Payne *et al.*, 2014)."

Colby COLBY COLLEGE
4000 Mayflower Hill
Waterville, Maine 04901
TEL 207-859-4000

• Introduction: The Smc5/6/Mms21 complex enhances the dissolution activity of the STR complex by promoting Sgs1 sumoylation, a process important for the assembly of the STR. - do you mean "important for STR complex dynamics".

Mms21 promotes sumoylation of Smc5/6 complex subunits and creates a binding platform for Sgs1's SIM to associate with the Smc5/6 complex (Bermudez-Lopez *et al.*, 2016). This brings the STR complex (Sgs1-Top3-Rmi1) in proximity to Mms21 and increases sumoylation of STR by Mms21 (Bermudez-Lopez *et al.*, 2016; Bonner *et al.*, 2016). Top3 binds sumoylated Sgs1 better, suggesting that sumoylation of Sgs1 by Mms21 promotes the assembly of the complex (Bonner *et al.*, 2016).

Given this context, we agree that "STR complex dynamics" would be more comprehensive to describe the process and modified the description in the introduction.

• Fig 3C: The authors study the progression of their untreated mutants from "Asyn into S phase". I am wondering why they did not first arrest their cells using alpha factor and then release them into the cell cycle. This seems like it may be a better way to study a potential cell cycle progression phenotype? Considering that the ∆22 mutants are HU sensitive, it could also be informative to see how the cell cycle profiles react in response to HU-induced arrest.

This is an excellent suggestion and we had a similar question, "Do *mms21∆* mutants traverse S-phase at the same rate as the wild-type?". Before submission of the manuscript, we have in fact attempted an experiment similar to the one suggested by the reviewer. We arrested cells using alpha factor and released them into MMS DNA damaging agent. The challenge was that even after 6 hours of alpha arrest, there was a considerable population of *mms21∆* mutants that remained in the G2/M phase. In addition, after 6 hours of arrest, cells started to escape the arrest although we replenished alpha factor every 1.5 hrs (an experimental practice descried in the literature such as (Biggins *et al.*, 2001) and (Cohen-Fix *et al.*, 1996)). We performed these experiments in *bar1∆* strains.

Thus, when the release into MMS was performed, it was extremely hard to interpret what percentage of the population moved into the S-phase after release, what percentage was already present before the release, or what percentage belatedly moved into the S-phase. Due to these observations, we decided to perform the current experiment presented in Figure 3C, demonstrating that *mms21∆* mutants exhibited prolonged G2/M.

• As stated, Mms21 is a subunit of the chromatin-associated Smc5/6 (structural maintenance of chromosome 5/6) complex that regulates DNA replication, repair, recombination, and chromosome segregation - do the authors test all of the above? I understand that the present analysis goes beyond the scope of this paper, but I think that a caveat needs to be added in the discussion that not all possible phenotypic assays associated with the ∆22 and human mutant were explored.

In the revised manuscript, we included the phenotypes that we did not examine in this study but will be informative to explore in the future. This description is in the discussion section (p.12).

Colby

COLBY COLLEGE
4000 Mayflower Hill
Waterville, Maine 04901
TEL 207-859-4000

"We acknowledge that the scope of our study is limited to genetic evidence, suggesting potential mechanisms by which Mms21's C-terminus contributes to genome stability. Our study did not examine whether the lack of the C-terminus disrupts DNA replication or recombination, processes known to be regulated by Mms21 and the Smc5/6 complex (Peng *et al.*, 2023). G2/M delay phenotypes associated with the C-terminal truncation in *mms21Δ22* mutants imply that these cells may experience replication stress and subsequent chromosome segregation errors. Likewise, we did not examine the localization of the C-terminally truncated Mms21 mutant. Direct biochemical or cellular assays are necessary to better understand the contributions of the C-terminus to Mms21's functions."

Reviewer #2 (Remarks to the Author):

Li et al. present a study examining the role of the c-terminus of the SUMO E3 ligase, Mms21. This protein has a conserved role in the maintenance of genome integrity as a component of the Smc5/6 complex, which is involved in resolving DNA intermediates following recombination. There is a rare genetic condition in humans caused by a mutation in the human homolog of Mms21, NSMCE2, that truncates the C-terminus of the protein. The authors created analogs to the human genes/proteins, mms21 Δ18, which ends at the same point as the human gene and lacks the C-terminal SIM present in the yeast gene, and mms21 Δ22, which ends at a point equivalent to the human disease mutant. They find that the human-like truncation (Δ18) is well tolerated in yeast, leading to no growth defects in the presence of DNA-damaging agents relative to WT Mms21. However, the disease mutant analog (Δ22) leads to compromised growth under the same DNA-damaging conditions. They find that yeast expressing the disease mutant have lower levels of mms21 protein, but that this does not appear to be the reason for their growth defects. Instead, it appears that the C-terminus both promotes ligase activity, and is also necessary for non-ligase activities of the protein.

The use of yeast Mms21 to investigate a human disease mutation is clever, and the work is carefully done, however there are problems that stop it from being suitable for publication in MBoC in its current form, primarily because the complex interplay of mutants and phenotypes used to derive the conclusions are difficult to follow, and so it needs significant rewriting to develop the clarity necessary. Some of this clarity in story telling may be facilitated by considering the predicted protein structure, which I think will improve the ways that the authors think and talk about their work. I believe the authors can likely revise the manuscript to address these concerns.

Major Issues
1. The authors should examine the alpha fold structure prediction for Mms21 (which is high confidence and has little IDR). The entire study makes sense in light of the predicted structure. The mms21 Δ18 truncation removes a c-terminal tail that sticks out from the structure. The residues that are present in Δ18, but removed in Δ22 contact both the N-terminus and the SP-RING domain, and appear to hold the whole protein together. Thus the disease mutant likely disrupts both SP-RING positioning or stability, as well as structural roles that Mms21 plays in the larger Smc5/6 complex. Incorporating an annotated structure to examine how the deleted regions are positioned relative to the N-terminus and the SP-RING would go a long way towards understanding what is happening here. Whether this is better at the beginning or the end, I leave

Colby
COLBY COLLEGE
4000 Mayflower Hill
Waterville, Maine 04901
TEL 207-859-4000

up to the authors. I could see advantages of either organization. Without this readily available information, the discussion feels unanchored. I think considering this structure would change that.

We appreciate this excellent suggestion to anchor the phenotypes of the mutants to the structure. Since the crystal structure of Smc5/Mms21 is available (PDB: 3HTK), we annotated this structure and included this as Figure 1D. We also tried the alpha fold structural prediction the sequences of *mms21Δ18* and *mms21Δ22* mutants. This prediction revealed the same message – four amino acids present in the *mms21Δ18* mutant, but not in the *mms21Δ22* mutant, are in proximity to the N-terminus and the SP-RING.

In the revised manuscript, we connected the structure information to the mutants studied at the beginning of the result section. In addition, we incorporated a discussion of how the structure informed us about the potential function of the C-terminus in influencing the ligase activity.

<u>In Result (p.5)</u>

"Using the published crystal structure of *S. cerevisiae* Mms21 bound to Smc5 (PDB ID: 3HTK), we visualized the location of those truncated residues within the structure (Duan *et al.*, 2009). The last 18 amino acids extend outwards from the core of the protein, ostensibly not accessible for intramolecular interactions with other regions of the protein (light red, Figure 1D). The four amino acids ("KIAK") present in the *mms21Δ18* mutant but not in the *mms21Δ22* mutant are flanked by the N-terminus and the SP-RING domain (dark red, Figure 1D). Structural information suggested that truncation of 22 amino acids was more likely to have an impact on the function of the protein."

<u>In Discussion (p.9)</u>

"The crystal structure of Mms21 bound to Smc5 reveals that the four amino acids ("KIAK") present in the benign truncation (*mms21Δ18)*, but not present in the disease allele analog (*mms21Δ22*), are in proximity to the N-terminus (Figure 1D). The N-terminus of Mms21 interacts with Smc5 (Duan *et al.*, 2009; Peng *et al.*, 2023). Thus, it is possible that the lack of KIAK residues in the disease allele analog destabilizes the Mms21-Smc5 interaction and impairs the ligase activity of Mms21. Another intriguing possibility is that the C-terminus regulation of the E3 ligase activity is crucial only in the context of chromatin-bound Smc5/6 complex when coordination among multiple regulatory pathways is necessary to tightly control the enzymatic activity of Mms21 inside the cell. In this model, the C-terminus may promote the function of the catalytically active SP-RING by scaffolding selective substrates. In an alternative scenario, the lack of KIAK residues close to the SP-RING domain may be one contributing factor that changes the conformation of the ligase domain and directly impair the catalytic activity of the enzyme (Figure 1D)."

2. The story is hard to follow. I believe part of this is the complexity of the mms21 mutant naming conventions, which are hard to keep track of. Descriptive text (such as I used above) when talking about mms21Δ18 vs mms21Δ22 would help the reader greatly. Another issue is that the authors set up a nice problem of the disease mutant in the introduction, but don't center their conclusions on that. They find that the truncation similar to WT human NSMCE2 does not disrupt function in yeast (at least appreciably), but that the disease mutant analog does. This framing is essentially

Colby

COLBY COLLEGE
4000 Mayflower Hill
Waterville, Maine 04901
TEL 207-859-4000

abandoned after the introduction, but is a very useful way to think about it. Successfully modeling a human disease mutant in yeast is great, the authors should make it more clear that they have succeeded in doing this.

We acknowledge that technical names of different mutants can disconnect the biological significance of the study from the experiments. Following the reviewer's advice, we introduced descriptive names of the alleles, such as "the disease allele analog" for the *mms21Δ22* mutant and "the benign truncation" for the *mms21Δ18* mutant in the revised manuscript. We labeled the cartoons with descriptive text so that a reader can use it as a quick reference (Figures 2A and 4A in the revised manuscript). Throughout the revised manuscript, we used descriptive text alongside technical names to center the take-home messages of the study on the biological significance of the mutant. Especially, we emphasized on the modeling of the disease allele analog in the discussion.

3. The explanation of the truncation effect on ligase-dependent function vs ligase-independent function was convoluted and required a lot of work on my part to understand. I ultimately came to the conclusion that the authors were warranted in their conclusions, but it was not easy. Given the centrality of this idea to their overall conclusions, this should be made much more clear. A cartoon summarizing the logic in this section might help.

We appreciate this suggestion to bring much better clarity to the argument. We restructured the organization of the entire sub-section of the result titled, "C-terminal truncation impairs ligase-dependent and -independent genome integrity functions of Mms21," so that the progression of logic becomes clear. Thus, Figure 4 in the revised manuscript was also reorganized.

In addition, we included a cartoon (Figure 4C in the revised manuscript) to describe the logic behind the rescue experiments, which provided evidence for the notion that the C-terminus contributed to ligase-dependent and -independent functions. Furthermore, we included Figure 5 in the revised manuscript to illustrate which mutants lacked the ligase-dependent function or both ligase-dependent and -independent functions.

4. With regards to the mutant nomenclature, a cartoon like the one in 4B would be helpful earlier in the paper, and containing all mutants used.

In the revised manuscript, we provided a cartoon in Figure 2A of the revised manuscript, depicting mutants used along with the description of the alleles, for a quick reference.

5. I don't find the missense analysis adds anything to the study. In fact, I think it serves as a distraction from the more relevant idea that you have a disease mutant that is informing your mutagenesis strategy. The missense analysis leads the reader to think there will examination of point mutants in I235, and that never happens. Instead it is ignored and they move back to the truncations that are justified by the human protein.

We agree with the reviewer that these mutants do not add to the story at this point. We removed this data from Figure 2A of the original manuscript.

Colby
COLBY COLLEGE
4000 Mayflower Hill
Waterville, Maine 04901
TEL 207-859-4000

6. The authors point out that the SP-RING is required for ligase activity, but is the SP-RING sufficient for ligase activity? Is the C-terminus a required part for ligase function in vitro, or is its requirement solely in the context of the larger Smc complex?

This is an excellent point. The literature suggests that Mms21's ligase activity may rely on additional factors beyond the SP-RING domain *in vivo*.

Due to technical limitations, we are unable to determine if the C-terminus is required for the ligase activity *in vivo* only in the context of the Smc5/6 complex. However, we agree with the reviewer that this is a crucial point to consider. Thus, we included a discussion of this point in the revised discussion (p.9).

"Evidence from the literature suggests that having an intact SP-RING domain is not sufficient, but additional regulations are needed for the optimal ligase activity of Mms21. The C-terminus may contribute to one of these regulatory pathways. For instance, ATPase activity and DNA binding of Smc5 enhance the SUMO ligase activity of Mms21 (Bermudez-Lopez *et al.*, 2015; Varejao *et al.*, 2018). The crystal structure of Mms21 bound to Smc5 reveals that the four amino acids ("KIAK") present in the benign truncation (*mms21Δ18)*, but not present in the disease allele analog (*mms21Δ22*), are in proximity to the N-terminus (Figure 1D). The N-terminus of Mms21 interacts with Smc5 (Duan *et al.*, 2009; Peng *et al.*, 2023). Thus, it is possible that the lack of KIAK residues in the disease allele analog destabilizes the Mms21-Smc5 interaction and impairs the ligase activity of Mms21. Another intriguing possibility is that the C-terminus regulation of the E3 ligase activity is crucial only in the context of chromatin-bound Smc5/6 complex when coordination among multiple regulatory pathways is necessary to tightly control the enzymatic activity of Mms21 inside the cell. In this model, the C-terminus may promote the function of the catalytically active SP-RING by scaffolding selective substrates."

7. Is the Δ22 dominant negative? The experiments with adding Δ22 on a plasmid do not test the impact of adding it to a WT background. The authors mention as a shortcoming that previous work only examined the human c-terminal truncation in the context of the WT protein. Testing this directly in yeast would add a lot to our understanding of the impact of this mutation.

Based on our genetic evidence, the *mms21Δ22* mutant did not behave as a dominant negative allele. In Figure 4D of the revised manuscript (Figure 4E of the original manuscript), we overexpressed the *mms21Δ22* mutant from pRS426 plasmid in the wild-type background. These cells exhibited growth and DNA damage sensitivity comparable to wild-type cells with the empty plasmid, suggesting that the *mms21Δ22* mutant did not interfere with the wild-type protein. We also described this as part of the discussion (p.11-12).

"In this model, the *mms21Δ22* mutant may act as a dominant negative mutant, preventing the Ubc9 E2 enzyme from working together with other E3 ligases, Siz1 and Siz2. However, our data suggest that negative effects through sequestration is unlikely. The endogenous expression level of the *mms21Δ22* mutant was significantly reduced to compete for Ubc9 against other proteins. Furthermore, when the *mms21Δ22* mutant was overexpressed in *MMS21* or *mms21-CH* background, cell growth and DNA damage sensitivity were comparable to cells without the

overexpression (Figure 4D). These data implied that the mutant, at least in the context of these experimental conditions, did not behave as a dominant negative mutant."

8. The problem solving with the integrating plasmid in the beginning is well done, but is a distraction from the scientific questions being asked. The authors may want to consider moving that section to the supplement, and covering what they did in broad strokes in a sentence or two and then move on to the experiments that effectively test Mms21 function.

Reviewer #1 also pointed out that we should move the figures regarding revertants to the supplemental materials. In the revised manuscript, as suggested by the reviewer, we summarized the data on revertants to support the main messages but moved the details of the description to the supplemental materials. Original Fig. 1D-G are now Suppl. Fig.1C-F.

To maintain the logical progression of the story, we included Fig2B in the revise manuscript to make comparison between *mms21-CH* and *mms21Δ22* mutants.

9. There is no reference to the number of replicates of the spotting assays.

This information is now included in the figure legends of the revised manuscript.

10. The western blotting used to assess mms21Δ22 levels should be quantified, analyzed, and the number of replicates listed in the figure legends.

We provided quantification of western blot analyses for *mms21Δ22* in comparison to wild-type and other mutants with statistical analyses. The number of replicates is included in the figure legends.

Minor Issues

1. The first "significance statement" doesn't read quite right, I think it's the middle sentence coupled with "to this end" at the beginning of the next. The other bullet points are fine, so I think the authors just need to iterate on this one.

We have revised the statement.

"•The SUMO E3 ligase function of an evolutionarily conserved Mms21 is crucial for genome integrity. The ligase-independent function of Mms21 in genome stability needs further exploration. To this end, we characterized a C-terminal truncation mutant of budding yeast Mms21 (*mms21Δ22*), analogous to a human mutation associated with genome instability conditions."

2. The time needed for arrest in alpha factor seems long. I assume these experiments were done in bar1Δ cells (especially given the low alpha factor concentration used)? There is mention of deleting BAR1 in the methods.

Colby

COLBY COLLEGE
4000 Mayflower Hill
Waterville, Maine 04901
TEL 207-859-4000

The time was relatively long since *mms21Δ22* mutants grew slowly. We observed that even after 6 hours of arrest, a considerable percentage of *mms21Δ22* mutants remained in G2/M.

Yes, the experiments were performed in *BAR1* knock out cells. We added a sentence in the results section to describe this. In addition, we also indicated that strains are *BAR1* knock out in the figure legend of Figure 3.

3. In the second-to-last paragraph in the results, the authors start with "In agreement with the data in Figures 1E and F..." This requires the reader to either remember that data, or stop and go back to look at the figure, when the authors could just say what their new experiments are agreeing with.

Since we moved the data on revertants (original Figures 1E and F), this description is no longer in the revised manuscript.

References

1. Bermudez-Lopez, M., Pocino-Merino, I., Sanchez, H., Bueno, A., Guasch, C., Almedawar, S., Bru-Virgili, S., Gari, E., Wyman, C., Reverter, D., *et al.* (2015). ATPase-dependent control of the Mms21 SUMO ligase during DNA repair. *PLoS Biol*, *13*(3), e1002089. https://doi.org/10.1371/journal.pbio.1002089
2. Bermudez-Lopez, M., Villoria, M. T., Esteras, M., Jarmuz, A., Torres-Rosell, J., Clemente-Blanco, A., & Aragon, L. (2016). Sgs1's roles in DNA end resection, HJ dissolution, and crossover suppression require a two-step SUMO regulation dependent on Smc5/6. *Genes Dev*, *30*(11), 1339-1356. https://doi.org/10.1101/gad.278275.116
3. Biggins, S., & Murray, A. W. (2001). The budding yeast protein kinase Ipl1/Aurora allows the absence of tension to activate the spindle checkpoint. *Genes Dev*, *15*(23), 3118-3129. https://doi.org/10.1101/gad.934801
4. Bonner, J. N., Choi, K., Xue, X., Torres, N. P., Szakal, B., Wei, L., Wan, B., Arter, M., Matos, J., Sung, P., *et al.* (2016). Smc5/6 Mediated Sumoylation of the Sgs1-Top3-Rmi1 Complex Promotes Removal of Recombination Intermediates. *Cell Rep*, *16*(2), 368-378. https://doi.org/10.1016/j.celrep.2016.06.015
5. Cohen-Fix, O., Peters, J. M., Kirschner, M. W., & Koshland, D. (1996). Anaphase initiation in Saccharomyces cerevisiae is controlled by the APC-dependent degradation of the anaphase inhibitor Pds1p. *Genes Dev*, *10*(24), 3081-3093. https://doi.org/10.1101/gad.10.24.3081
6. Duan, X., Sarangi, P., Liu, X., Rangi, G. K., Zhao, X., & Ye, H. (2009). Structural and functional insights into the roles of the Mms21 subunit of the Smc5/6 complex. *Mol Cell*, *35*(5), 657-668. https://doi.org/10.1016/j.molcel.2009.06.032
7. Payne, F., Colnaghi, R., Rocha, N., Seth, A., Harris, J., Carpenter, G., Bottomley, W. E., Wheeler, E., Wong, S., Saudek, V., *et al.* (2014). Hypomorphism in human NSMCE2 linked to primordial dwarfism and insulin resistance. *J Clin Invest*, *124*(9), 4028-4038. https://doi.org/10.1172/JCI73264
8. Peng, X. P., & Zhao, X. (2023). The multi-functional Smc5/6 complex in genome protection and disease. *Nat Struct Mol Biol*, *30*(6), 724-734. https://doi.org/10.1038/s41594-023-01015-6

Colby
COLBY COLLEGE
4000 Mayflower Hill
Waterville, Maine 04901
TEL 207-859-4000

9. Thu, Y. M., Van Riper, S. K., Higgins, L., Zhang, T., Becker, J. R., Markowski, T. W., Nguyen, H. D., Griffin, T. J., & Bielinsky, A. K. (2016). Slx5/Slx8 Promotes Replication Stress Tolerance by Facilitating Mitotic Progression. *Cell Rep*, *15*(6), 1254-1265. https://doi.org/10.1016/j.celrep.2016.04.017
10. Varejao, N., Ibars, E., Lascorz, J., Colomina, N., Torres-Rosell, J., & Reverter, D. (2018). DNA activates the Nse2/Mms21 SUMO E3 ligase in the Smc5/6 complex. *EMBO J*, *37*(12). https://doi.org/10.15252/embj.201798306

RE: Manuscript #E25-11-0567R
TITLE: "Ligase-dependent and independent functions of the C-terminus of Mms21 contribute to optimal growth and genome stability in Saccharomyces cerevisiae"

Dear Dr. Thu:

I am pleased to accept your manuscript for publication in Molecular Biology of the Cell.

Sincerely,
Richard Wozniak
Monitoring Editor
Molecular Biology of the Cell

Dear Dr. Thu:

Congratulations on the acceptance of your manuscript! Thank you for publishing your work in Molecular Biology of the Cell (MBoC).

Within 10 days, an unedited PDF of your manuscript will be published on MBoC in Press, an early-release journal version. The date your manuscript appears on this site is the official publication date.

Your copyedited and typeset manuscript will be scheduled for publication in the next available issue of MBoC. Once your paper is ready for review, our production team will notify you. In the summer of 2024, our production provider will introduce an online proofing solution, which will streamline the process of checking and validating author changes. This will result in improved typesetting quality, enhanced digital content, and a faster overall process.

We invite you to submit images related to your accepted manuscript to be considered for the journal cover.
Cover images must contain color, TIF Format
Size image at 7.4" wide x 5.85" deep
RGB and CMYK color (please submit two separate files, one in each format)
300 dpi resolution

Please also send a 1-paragraph description of the image that a general audience could understand. Include with the caption the name and institution of the individual(s) the image should be attributed to. Please provide this description in a Word file. You can send the file via email or by your favorite file transfer service to mboc@ascb.org.

Authors of Articles and Brief Communications are also encouraged to create a short video abstract to accompany their article when it is published. These video abstracts, known as Science Sketches, are up to 2 minutes long and will be published on YouTube and then embedded in the article abstract. Science Sketch Editors on the MBoC Editorial Board will guide you as you prepare your video. Information about how to prepare and submit a video abstract is available at www.molbiolcell.org/science-sketches. Please contact mboc@ascb.org if you are interested in creating a Science Sketch.

We look forward to publishing your paper in MBoC.
Sign up for MBoC's new content alerts here https://tinyurl.com/3bcsyk3e

Sincerely,

Eric Baker
Journal Production Manager
MBoC Editorial Office
mbc@ascb.org

Reviewer #1 (Remarks to the Author):

The manuscript has been edited and improved through writing and organization. Some requested caveats were included. Although no additional Δ22 mutant data (e.g localization, nucleolar morphology, or sumoylation analysis) were included during revision, I stand by my statement that, while ultimately this study falls short on explaining the phenotype of the Δ22 mutants, this study is an important resource and step towards a better understanding of Mms21's structure and function. Minor: mms21 22 mutants exhibited slower growth and DNA damage sensitivity than the wild-type ... - should be slower growth and increased DNA damage sensitivity compared to the wildtype

Reviewer #2 (Remarks to the Author):

The authors have dealt with all my concerns. My only remaining feedback is that the authors may want to consider, when they say "The phenotypic similarities between mms21 22 and mms21-
CH 22 mutants validated this idea - negative phenotypes associated with the unregulated SPAR region persisted when the SP-RING domain was mutated (Figures 4B and 5). Finally, our genetic evidence suggested that the ligase-independent function of the C-terminus is to tightly control the function of the SPAR region. Improved growth and DNA damage sensitivity of mms21-CH mutants compared to mms21 22 mutants and the partial rescue of mms21 22 cells by the mms21-CH mutant supported this conclusion - the intact C-terminus could restrain the SPAR independently of the ligase function (Figures 2B, 4E, 4F, and 5)."

Does their evidence suggest that the c-terminus controls function of the SPAR region, or is it necessary for the SPAR region to function (fold?)? Both scenarios might lead to the same outcome, but I think the data at hand may not argue for regulation over structural requirements.

Additionally, I note that the affiliation of the first author has changed since the first submission. While the authors can deal with this how they want, I think it would be standard practice for the affiliation to be with the institution where the work was done, even if the author has now moved on to a new institution.
